# *Lactobacillus johnsonii* N6.2 phospholipids induce immature-like dendritic cells with a migratory-regulatory-like transcriptional signature

**DOI:** 10.1080/19490976.2023.2252447

**Published:** 2023-09-07

**Authors:** Alexandra E. Cuaycal, Leandro Dias Teixeira, Graciela L. Lorca, Claudio F. Gonzalez

**Affiliations:** Department of Microbiology and Cell Science, Genetics Institute, Institute of Food and Agricultural Sciences, University of Florida, Gainesville, FL, USA

**Keywords:** *Lactobacillus johnsonii* N6.2, probiotic, microbial-derived lipids, phospholipid, innate immunity, dendritic cell, cellular immune response, immunomodulation

## Abstract

Shifts in the gut microbiota composition, called dysbiosis, have been directly associated with acute and chronic diseases. However, the underlying biological systems connecting gut dysbiosis to systemic inflammatory pathologies are not well understood. Phospholipids (PLs) act as precursors of both, bioactive inflammatory and resolving mediators. Their dysregulation is associated with chronic diseases including cancer. Gut microbial-derived lipids are structurally unique and capable of modulating host’s immunity. *Lactobacillus johnsonii* N6.2 is a Gram-positive gut symbiont with probiotic characteristics. *L. johnsonii* N6.2 reduces the incidence of autoimmunity in animal models of Type 1 Diabetes and improves general wellness in healthy volunteers by promoting, in part, local and systemic anti-inflammatory responses. By utilizing bioassay-guided fractionation methods with bone marrow-derived dendritic cells (BMDCs), we report here that *L. johnsonii* N6.2 purified lipids induce a transcriptional signature that resembles that of migratory (mig) DCs. RNAseq-based analysis showed that BMDCs stimulated with *L. johnsonii N6.2* total lipids upregulate maturation-mig related genes *Cd86*, *Cd40*, *Ccr7*, *Icam1* along with immunoregulatory genes including *Itgb8*, *Nfkbiz*, *Jag1*, *Adora2a*, *IL2ra*, *Arg1*, and *Cd274*. Quantitative reverse transcription (qRT)-PCR analysis indicated that PLs are the bioactive lipids triggering the BMDCs response. Antibody-blocking of surface Toll-like receptor (TLR)2 resulted in boosted PL-mediated upregulation of pro-inflammatory *Il6*. Chemical inhibition of the IKKα kinase from the non-canonical NF-κB pathway specifically restricted upregulation of *Il6* and *Tnf*. Phenotypically, PL-stimulated BMDCs displayed an immature like-phenotype with significantly increased surface ICAM-1. This study provides insight into the immunoregulatory capacity of Gram-positive, gut microbial-derived phospholipids on innate immune responses.

## Introduction

Alterations in bacterial diversity lead to microbiota structural imbalances in the gut called dysbiosis. Important shifts in the microbiota composition have been extensively documented and directly associated with acute and chronic diseases.^1–3^ But the underlying biological systems explaining such correlations are poorly described. Certainly, dysbiosis has been attributed as a major cause of several inflammatory and autoimmune disorders, including diabetes, inflammatory bowel disease (IBD), and rheumatoid arthritis. However, the mechanisms connecting gut dysbiosis and systemic effects linked to such specific pathologies are not well understood.^4^ Gut dysbiosis can be corrected by using fecal transplant obtained from healthy donors, as well as by using therapeutic probiotics.^5^ Specific bacterial strains used as probiotics can contribute to restore the gut microbiota balance and modulate the immune response. Several microbial-derived bioactive metabolites such as short-chain fatty acids (SCFAs), tryptophan metabolites, and secondary bile acids diffuse through the mucosa, interact with specific receptors and regulate host’s physiology and immunity.^6–8^

The immune system surveillance in the gut epithelium is mainly performed by antigen presenting cells (APCs), also called dendritic cells (DCs). These specialized cells work as a direct link between innate and adaptive immune responses. After up taking a new antigen (Ag), DCs intracellularly process it and present it to T cells in the draining lymph nodes (LNs), triggering the activation of adaptive immunity. Thus, DCs are distributed in both lymphoid and non-lymphoid tissues, displaying great heterogeneity regarding their functional and maturation state.^9^ While DCs present peptide Ags and activate conventional T cells for the initiation of adaptive immune responses, they also control the tolerogenic response, and as such, are key modulators of the host’s overall immunity.^10^ DCs can sense, process, and present lipid Ags through their CD1d molecules, for the stimulation of invariant NKT (iNKT) cells and subsequent modulation of immune responses.^11^ In the gut, DCs are distributed throughout the intestinal mucosa and play a central role in maintaining intestinal immune homeostasis, as they are the sentinel cells that control both innate and adaptive immunity in such an Ag-diverse environment.^12^

Lipids are increasingly being recognized for their signaling roles in fundamental cellular processes such as cell proliferation, survival, apoptosis, and cell metabolism. In fact, they are key bioactive mediators of inflammatory responses, and their dysregulation is involved in the development of chronic diseases including rheumatoid arthritis, inflammatory bowel disease, diabetes, and cancer.^13,14^ Structurally, they are highly diverse molecules with over 40,000 species, divided into eight basic categories according to their structure.^15^ Particularly, phospholipids are a diverse group of lipids with important signaling properties. Endogenous phospholipids act as precursors of either bioactive lipid mediators of inflammation (mainly eicosanoids) or of bioactive resolving mediators of inflammatory responses (lysophospholipids and endocannabinoids).^14^ While endogenous signaling phospholipids have been widely studied and their indispensable roles in tissue homeostasis are actively being determined, little is known about the involvement of gut commensal-derived lipids in the modulation of host’s immunity.

We recently reported the immunological properties of *Lactobacillus johnsonii* nanovesicles isolated directly from the bacterial culture supernatants.^16^ *L. johnsonii* N6.2 nanovesicles, composed of a complex lipid profile, surface proteins, and nucleic acids, were able to induce a strong innate immune response *in vitro*.^17^ Those *in vitro* results showed a direct correlation with the presence of IgA and IgG antibodies in human blood samples from healthy adults consuming the probiotic.^16^ Nonetheless, the specific components of these complex nanovesicles prompting the immune response were not yet identified.

Unique, gut-microbial derived lipids have been recently characterized in the murine intestine.^18^ Strikingly, these microbial-derived lipids are structurally different from host’s endogenous lipids and are emerging as active modulators of host’s immunity.^19–21^ The involvement of sphingolipids, specifically α-galactosylceramides (α-GalCers), derived from the Gram-negative bacteria and gut symbionts *Bacterioides* spp., on the homeostasis of gut immune cell is well documented.^19,22–24^ Recently, phospholipids and ornithine lipids derived from *Akkermansia muciniphila* were described as modulators of the innate immune response.^21,25^ Indeed, the participation of microbial components (including bacterial-derived lipids) in maintenance of host’s health has been described for several species of the *Bacteroidetes* and *Verrucomicrobia* families.^21,25–28^ Undoubtedly, the involvement of bacterio-lipids in host’s gut homeostasis is driving a strong scientific interest.

The central goal of the present article was to evaluate the immune-stimulating properties of lipids synthesized by *L. johnsonii* N6.2, a probiotic strain with proven beneficial effects on humans’ immune system.^29^ *L. johnsonii* N6.2 is a gut symbiont isolated from the gut of diabetic resistant rats. This bacterial strain presents probiotic characteristics and has been demonstrated to 1) delay the onset of Type 1 Diabetes (T1D) in diabetic prone (BBDP) rats, 2) modulate host’s phospholipid dynamics under a high fat diet and 3) improve general wellness in healthy adults.^29–31^ *L. johnsonii* N6.2 exerts its effect, in part, by interacting with the innate and adaptive immune system; inducing a Th17 cell bias in mesenteric lymph nodes of BBDP rats and modulating innate and adaptive immune cell populations in healthy volunteers.^29,32^ By combining transcriptional and phenotypical analyses in cell-based assays, we report here that *L. johnsonii* N6.2 phospholipids are immunogenic and induce a migratory-regulatory-like transcriptional signature in BMDCs.

## Results

### Purified lipids induce a gene expression pattern in bone marrow-derived dendritic cells

**Figure 1.**
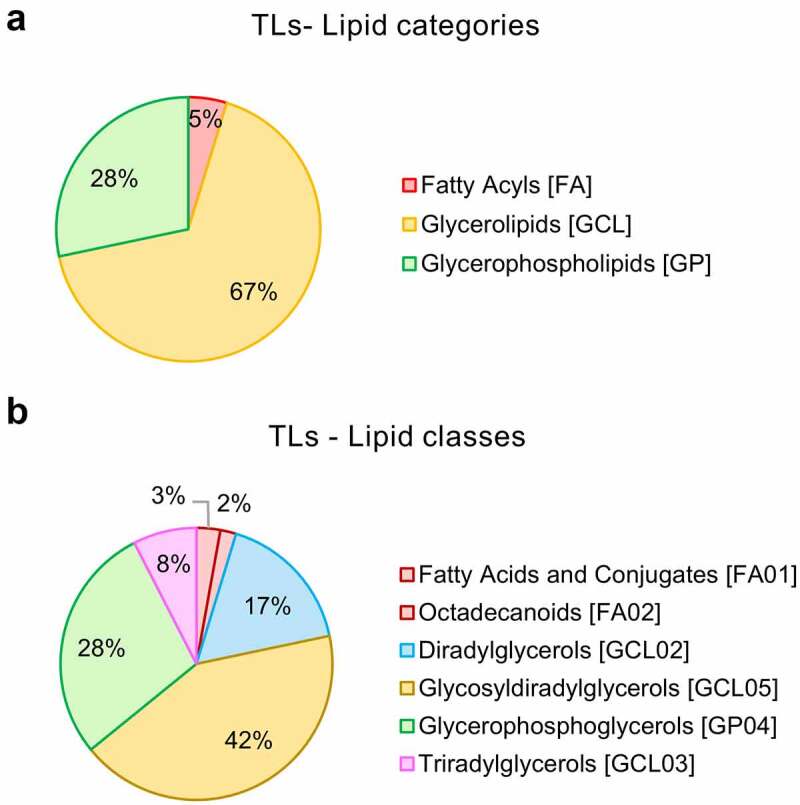
Global lipidomics analysis of *Lactobacillus johnsonii* N6.2 total lipids (TL). Total lipids from *L. johnsonii* N6.2 were purified and pooled from a large-scale culture and profiled by qualitative LC-MS/MS analysis. a: Percentage abundances in lipid categories. b: Percentage abundances in lipid classes. The abbreviation GCL for glycerolipids was selected to avoid confusion with GL (denotation used for the glycolipid fraction obtained after lipid fractionation of *L. johnsonii* N6.2 total lipids).

Qualitative lipidomic profiling of *L. johnsonii* N6.2-purified lipids revealed that total lipids (TLs) from *L. johnsonii* N6.2 are comprised of 67% glycerolipids (as glycosyldiradylglycerols (42%), diradylglycerols (17%), and triradylglycerols (8%)); 28% glycerophospholipids (as glyerophosphoglycerols) and 5% fatty acids (including 3% of fatty acids and conjugates and 2% octadecanoids) (Figure 1a,b). The lipidomic features detected for *L. johnsonii* N6.2 TLs as spectral (MS-2) annotations are displayed in Table S1. A total of 106 annotations were identified by spectral matching, representing only 6% of total readouts (see Table S2).

To determine the immune-stimulating properties of *L. johnsonii* N6.2-purified lipids, we generated rat BMDCs and stimulated the cultures with TLs. Cytotoxicity of the purified lipids was assessed by the 3-(4,5-dimethylthiazol-2-yl)-2,5-diphenyltetrazolium bromide (MTT) assay. No significant detrimental effects were found (Figure S1a). Complementary, cell viability was also assessed by flow cytometry during each assay and determined to consistently be over 95% (Figure S1b). The lipid samples were used as a homogeneous emulsion in PBS buffer pH 7.2. This assay was carried out in 7-day-old cultures having 10^6^ BMDCs per treatment. The cells were stimulated for 6 h with 600 ng/mL of *L. johnsonii* N6.2 TLs. The TL concentration tested was previously titrated and selected based on its stimulatory capability as determined by gene expression changes (Figure S2). We measured the induction of *Il6, Il10* and *Tnf* to evaluate the stimulatory capacity of the TLs obtained (Figure S2a) and posteriorly, expression of *Il6* and *Il10* for each lipid fraction assayed (Figure S2b). A minimum stimulation period of 6 h was determined to be enough to reproducibly follow the changes in gene expression among TLs and the fractions. In addition, stimulations with TLs from representative Gram-positive and Gram-negative bacteria showed that *L. johnsonii* N6.2 TLs induce a significantly distinct gene expression profile for the genes evaluated (Figure S3).

**Figure 2.**
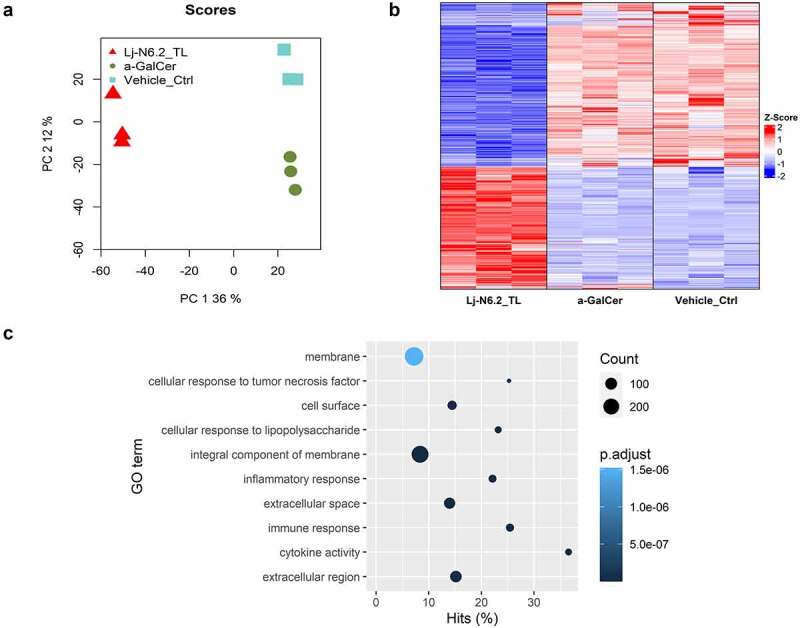
RNAseq-based global evaluation of BMDCs responses to total lipids derived from *Lactobacillus johnsonii* N6.2. a: Principal component analysis of treatment groups (Lj-N6.2_TL: *L. johnsonii* N6.2-TL-treated group; a-GalCer: α-GalCer-treated group; and Vehicle_control). b: Heatmap of total significantly differentially expressed genes (DEG) in Lj-N6.2_TL group (p.adjust < 0.01). The data showed a specific expression signature induced by total lipids from *L. johnsonii* N6.2 on BMDCs. c: Gene ontology enrichment analysis of DEG in *L.* *johnsonii* N6.2 treatment with absolute values of log2FC ≥ 1. Data represent three independent assays.

We used 100 ng/mL of synthetic α-GalCer (KRN700) or the vehicle control to compare the global stimulation patterns obtained with *L. johnsonii* N6.2 TLs. RNAseq-based analysis demonstrates that stimulation of BMDCs with TLs induces a distinct pattern of gene expression. We identified 2515 significantly differentially expressed genes (DEGs) (p.adjust < 0.01, |log2FC| > 0.3) when compared to the prototypical α-GalCer. When the samples were compared to the vehicle control (Figure 2a,b), 2283 genes exhibited differential expression (p.adjust < 0.01, |log2FC| > 0.3, Table S3). From this group of DEGs, we selected *Il6, Il10, Tlr2, Tlr4*, Adora2a *and Cyp2s1* as representative genes of TL-response, to be analyzed in the subsequent assays performed. Thus, we used the expression pattern of those selected genes as quality control to certify that the BMDCs generated in each batch will display similar responses in all subsequent assays. The same genes were used to evaluate the effects of the lipid fractions separated by chromatographic methods. In all cases, the expression of these response-markers was monitored by qRT-PCR.

To rule out possible *L. johnsonii* N6.2 exopolysaccharide (EPS) contamination in our lipid extractions, we stimulated BMDCs with EPS extracted from the same strain. The polymer was extracted from both the cell culture medium and that associated with bacterial cells. Our results showed that BMDCs stimulated with TLs from *L. johnsonii* N6.2 significantly upregulate *Il6, Tlr2*, and *Adora2a*; and downregulate *Tlr4* and *Cyp2s1* to a greater magnitude when compared to EPS from the same bacteria. Both EPS and TLs significantly induce *Il10* at similar levels (Figure S4).

### *L.* *johnsonii* N6.2 lipids suppress the inflammatory response

Given the extent of significant DEGs, we conducted Gene Ontology (GO) enrichment analysis using DEGs with an absolute log2FC ≥ 1 (719 DEGs). Figure 2c shows the top 10 significantly enriched GO terms (p.adjust < 0.01, for a full list see Table S4). BMDCs exposed to *L. johnsonii* N6.2 TLs are responding to the stimulus as it was expected. The significant biological processes affected (BPs) corresponded to: “immune response” (GO:0006955), “inflammatory response” (GO:0006954), “cellular response to tumor necrosis factor” (GO:0071356) and interestingly, “cellular response to lipopolysaccharide” (GO:0071222). These results are intriguing because *L. johnsonii* N6.2 is a Gram-positive bacterium that does not produce LPS. Furthermore, “response to bacterium” (GO:0009617) and “response to molecule of bacterial origin” (GO:0002237) were also enriched (p.adjust < 0.01, Table S4). Remarkably, the cellular components (CCs) that corresponded to “extracellular region” (GO:0005576), “extracellular space” (GO:0005615), “integral component of membrane” (GO:0016021), “cell surface” (GO:0009986) and “membrane” (GO:0016020) (Figure 2c) were also over-stimulated by the treatment.

The results also showed 14 GO terms of positive regulation and 8 GOs of negative regulation. Interestingly, the negatively regulated group is composed of several BPs related to immune/inflammatory responses (see Table S4). Interferon-gamma, tumor necrosis factor, interleukin-12, NF-kappaB transcription factor activity, and the inflammatory response (see Figure S5a) were classified in this negatively regulated group. The top 10 BPs of positive regulation included cytokine production, angiogenesis-related genes, and the peptidyl-tyrosine phosphorylation pathway (see Figure S5b).

The GO enrichment analysis altogether with the results obtained with the RNAseq-based exploratory assay and pair-wise differential expression analyses suggest that TLs from *L. johnsonii* N6.2 exert a transcriptional signature upon stimulation of BMDCs.

### BMDC maturation and regulatory factors are upregulated after TLs induction

**Figure 3.**
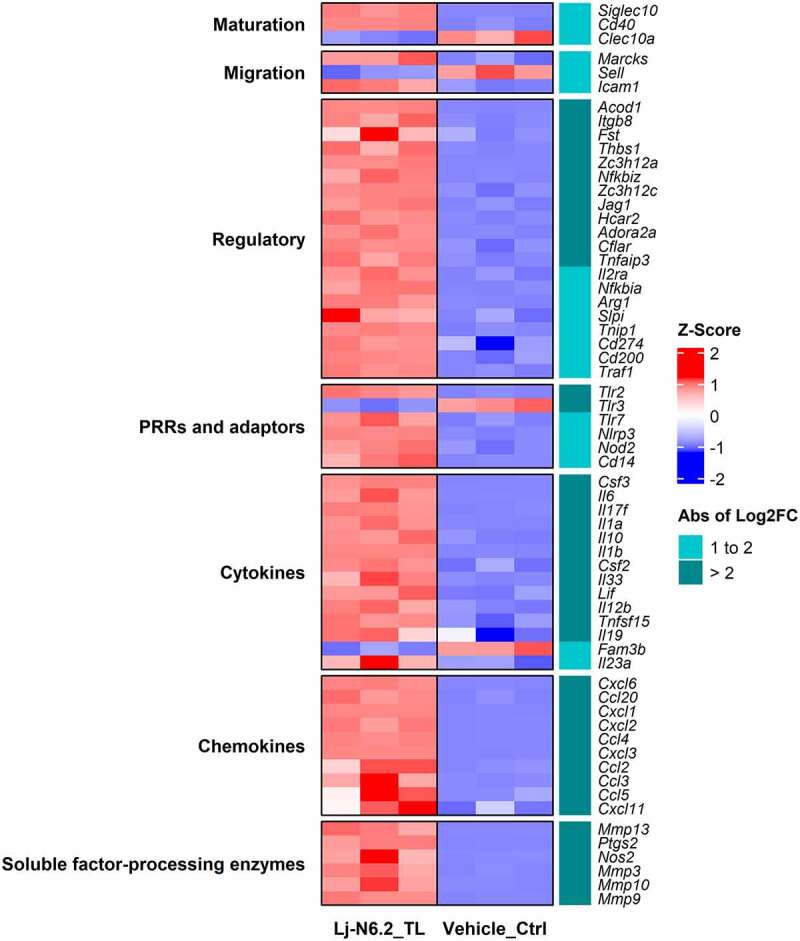
RNAseq-based analysis of BMDCs response to total lipids derived from *L. johnsonii* N6.2. Heatmap displays selected DEGs with absolute values of log2FC between 1 and 2 and greater than 2 (p.adjust < 0.01). Total lipids (TLs) from *L. johnsonii* N6.2 significantly upregulate several regulatory molecules involved in the regulation and suppression of immune responses. The graphic displays the results obtained from three independent assays.

To perform a deeper analysis of the BMDCs response, we selected DEGs with a transcriptional change higher than |log2FC| > 2; a total of 293 were included in this group. Figure 3 displays the expression patterns of selected DEGs; the figure includes positively and negatively regulated genes.

DCs stimulated with TLs from *L. johnsonii* N6.2 induced transcriptional upregulation of cytokines and chemokines, including *Il6, Il17f, Il1a, Il1b, Il12b, Il23a, Ccl2–5, Cxcl1–3, Cxcl6, Cxcl20*, and the anti-inflammatory cytokine *Il10*. High induction of hematopoietic growth factors such as colony stimulating factor 2 and 3 (*Csf2, Csf3*) was also observed. Pattern recognition receptors (PRR) such as Toll-like receptor *(Tlr)-2*, *Tlr7* and Nucleotide-binding oligomerization domain 2 (*Nod2*) were also induced. Interestingly, *Tlr3* and *Tlr4* were downregulated. Matrix metalloproteases *Mmp3, Mmp9, Mmp10*, and *Mmp13*, known to process and regulate the extracellular milieu of soluble factors, were highly upregulated (logFC >2) along with the prostaglandin E2-producing enzyme *Ptgs2* and inducible nitric oxide synthase (iNOS, gene *Nos2*).

Intriguingly, *L. johnsonii* N6.2 TLs induced the expression of *Siglec10* while downregulating *Clec10a*; both markers characteristic of the immature state.^33^ Furthermore, the BMDCs displayed weak to moderate upregulation of co-stimulatory molecules *Cd40*, *Cd86*, and downregulation of *Cd83*. These genes are associated with DC maturation. Nonetheless, moderate upregulation of migration markers *Marcks, Icam1, Ccr7*, and *Fscn1* along with moderate downregulation of L-selectin or CD62L (gene *Sell*, Log2FC = −1.5) was also observed. These molecules are important for the migration of DCs present in nonlymphoid-tissues to the draining lymph nodes to regulate T-cell immunity.^9,34^

*L. johnsonii* N6.2-TL stimulation induced high upregulation of a selected group of immunoregulatory molecules. This includes direct or indirect T cell suppressors (Figure 3 – Regulatory category). As such, upregulation of *Adora2a* (encoding the adenosine A2A receptor), *Jag1* (encoding Jagged-1, a Notch ligand), *Itgb8* (encoding the integrin subunit beta 8), *Zc3h12a* (encoding the endonuclease Regnase-1), *Hcar2* (encoding the hydroxycarboxylic acid receptor 2 or GPR109A) and negative regulators of Nf-κB signaling was observed. This is associated to the upregulation of *Arg1, Cd200, Il2ra* (encoding CD25), *Cd274* (encoding PD-L1). Other regulatory molecules such as *Il4i1, Aldh1a2*, and the suppressor of cytokine signaling (*Socs*) *1* showed lower levels of induction. Interestingly, *Fam3b*, a pro-apoptotic cytokine associated with diabetogenic pancreatic cells,^35^ was highly downregulated (Log2FC = −2.2). This result correlates with the ability of this strain to mitigate the incidence of T1D in Bio-Breeding diabetic prone rats (a rat strain that spontaneously develops a T-cell dependent autoimmune disease comparable to T1D in humans) as previously published.^30^

Transcriptomic analyses of DC subsets have revealed that DCs undergoing maturation and activation display a similar transcriptional signature regardless of their ontology. Similarly, steady-state migratory-like DCs also share a particular signature of immunoregulatory genes irrespective of their tissue or lineage origin.^9,36^ Rat BMDCs generated with CSF2/granulocyte/macrophage colony-stimulating factor (GM-CSF) and FLT3L represent a heterogeneous population that comprises mainly of classical (c)DCs and some plasmacytoid (p)DCs.^37^ Indeed, the expression of classical-related genes for these DC populations was varied in our transcriptomic analysis (see Figure S6). We observed upregulation of the cDC-related genes: *Itgax* (encoding CD11c) and *Glipr2* along with downregulation of other cDC-related genes such as *Ifi30*, *Ly86, Entpd1*, and *Siglec1*. Nonetheless, classical cDC2-related genes *Itgam* (encoding CD11b) and *Nfil3* were upregulated while cDC1-related genes *Irf8*, *Itgae* and the typical pDC-related gene *Ptcra* were downregulated.^38,39^ We also observed downregulation of Clec10a, and upregulation of the monocyte-related gene CD14. These results suggest that the dendritic cells are polarized toward the cDC2-like subsets cDC2A and DC3, respectively.^38,39^

In summary, our analysis of expression patterns allowed us to conclude that BMDCs stimulated with *L. johnsonii* N6.2 TLs displayed a cDC2-related transcriptomic profile with a migratory-regulatory-like transcriptional signature.

### Purified phospholipids induce a similar gene expression than TLs

TLs from *L. johnsonii* N6.2 are comprised of glycerolipids (glycosyldiradylglycerols, diradylglycerols and triradylglycerols), glycerophospholipids, and fatty acids as determined by mass spectroscopy (MS-2) (Figure 1 and Table S1). We have utilized a bioassay-guided fractionation to further separate *L. johnsonii* N6.2-purified lipids and determine the bioactive lipid fraction with the capability to stimulate DCs. We obtained the following fractions: simple lipids (SL, nonpolar and fatty acids), glycolipids (GL), and phospholipids (PL) by silica gel column chromatography. The fractionation was performed using the modified Frostegård method described by Dickson et al.^40^ Analysis of the fractions (SL, GL, and PL) revealed that the method produced only negligible amounts of carryover of lipid species when the fractions were compared (see Figure S7a).

**Figure 4.**
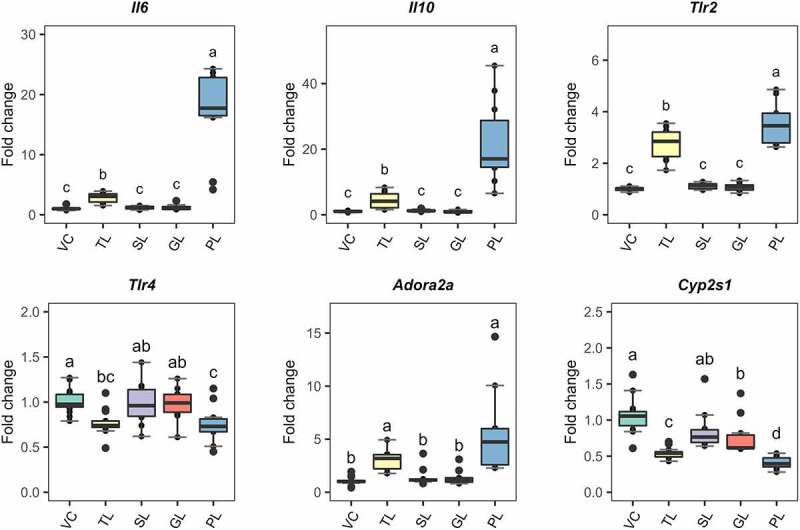
Gene expression of selected response-markers after fractionation of *L. johnsonii* N6.2 lipids into simple lipids (SL), glycolipids (GL) and phospholipids (PL). BMDCs were stimulated with total lipids (TL) at 500 ng/mL or three different fractions, at 5 µg/mL (SL, GL, and PL) for 6 h. VC: Vehicle control. The graphic displays the results obtained from three independent assays. Different letter labels denote statistically significant changes (p.value < 0.05).

These fractions were used to challenge DCs using identical procedures to those described for the RNAseq-based experiment. 7-day BMDCs were stimulated during 6 h with either 0.5 µg/mL of TLs (as a control) or 5 µg/mL of the respective lipid fractions. These concentrations were selected to ensure that the cells would get sufficient stimulation to detect reproducible changes in gene expression with qRT-PCR. To compare the effects of each fraction, we quantified the expression of *Il6, Il10, Tlr2*, and Adora2a (Figure 4). We also included *Tlr4* and *Cyp2s1*; these genes were downregulated in the RNAseq analysis. The PL fraction resulted in significant differential expression (p.value < 0.05) of the selected response-markers. The impact on the gene transcription was higher or similar when the values were compared to those observed using TLs. As such, PLs from *L. johnsonii* N6.2 induced significant upregulation of *Il6, Il10, and Tlr2* at a higher magnitude when compared to TLs and greater downregulation of *Cyp2s1*. Similar changes were observed for *Tlr4* and *Adora2a*. The GL fraction induced significant downregulation of *Cyp2s1* but at lower extent when compared to TLs and the PL fraction.

### *L.* *johnsonii* N6.2 phospholipids stimulate an immature-migratory-like phenotype in BMDCs

Once DCs are exposed to Ags, DCs undergo phenotypic changes to increase their surface expression of MHC class I (MHC-I) and MHC class II (MHC-II), costimulatory molecules and cell migration molecules. As such, mature DCs are capable of migrating to draining lymph nodes for the efficient Ag presentation and initiation of T-cell immunity.^34,41^ Given that BMDCs stimulated with *L. johnsonii* N6.2 TLs highly upregulate gene expression of immunoregulatory molecules, we set out to determine their maturation state by measuring the surface expression of MHC-II, costimulatory molecules CD80, CD86 and CD40 and the migration marker ICAM-1 after *L. johnsonii* N6.2 TL- and lipid fractions stimulations.

**Figure 5.**
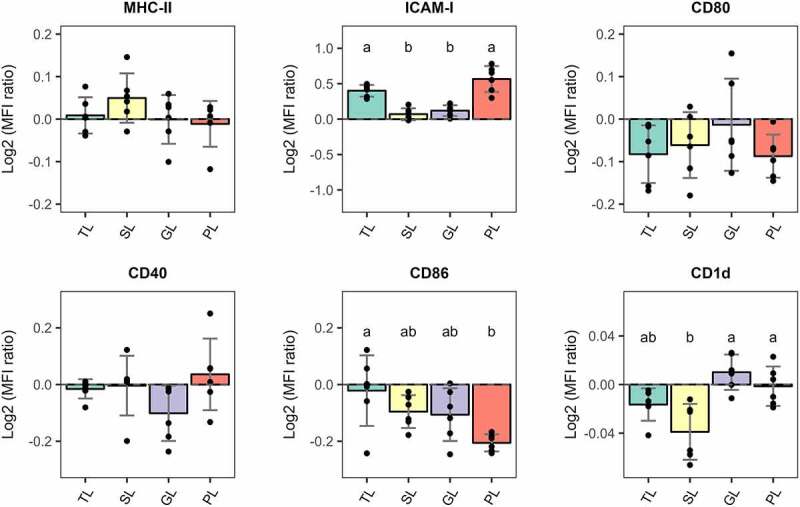
Relative surface expression of maturation and migration markers in BMDCs normalized to vehicle-treated cells and presented as Log2 values. BMDCs were stimulated with total lipids (TLs) at 500 ng/mL or fractions at 5 µg/mL for 6 h. SL: simple lipids, GL: glycolipids, PL: phospholipids. PLs significantly reduce surface expression of CD86 and induce a sig. increase of migration marker ICAM-1. VC: Vehicle control. The graphic displays the results obtained from three independent assays. Different letter labels denote statistically significant changes (p.value < 0.05).

To this end, BMDCs were stimulated with *L. johnsonii* N6.2 TLs or fractionated lipids (SL, GL, and PL) as described above and the levels of maturation markers on the cell surface were determined by flow cytometry. The fluorescence intensity levels for each marker are presented in Figure 5 as a log2 value of the ratio between the median fluorescence intensities (MFI) for each lipid stimulation (TL, SL, GL, and PL) and the MFI levels of the vehicle control-treated cells. The histogram overlays corresponding to significant changes are shown in Figure S8. Remarkably, the surface expression of CD86 was significantly and exclusively decreased (p.value < 0.05) only on BMDCs stimulated with *L. johnsonii* N6.2 PLs and no changes in the surface levels of MHC-II, CD80 and CD40 molecules were observed. Interestingly, the migration marker ICAM-1 was significantly and comparably increased (p.value < 0.05) on the surface of BMDCs stimulated with either *L. johnsonii* N6.2 TLs or PLs. Furthermore, the surface expression levels of the CD1d molecule, a non-polymorphic MHC-I-like transmembrane molecule that presents antigenic lipids to iNKT cells, was significantly decreased on SL-stimulated cells when compared to GL and PL-stimulated BMDCs.

Overall, BMDCs stimulated with *L. johnsonii* N6.2 PLs for 6 h displayed an immature-migratory-like phenotype with no increase in the surface expression levels of MHC-II, and costimulatory molecules CD80, CD86, and CD40, but higher levels of the migratory marker ICAM-1.

### Blockage of toll-like receptor 2 (TLR2) during BMDCs stimulation with phospholipids

After BMDC stimulation with *L. johnsonii* N6.2 TLs, the expression of *Tlr2* was significantly upregulated when compared to other Toll-like receptors (TLRs) (Figure 3). To confirm the involvement of TLR2 in the response observed, we blocked the receptors on the surface of BMDCs with monoclonal anti-TLR-2 antibody (Ab). BMDCs were treated with anti-TLR2 and the isotype control Abs using a concentration of 9 µg/mL during 1 h before addition of *L. johnsonii* N6.2 PLs and further incubation for 6 more hours. As negative controls, cells were stimulated with the vehicle control (for PLs), the Ab storage buffer and both combined. BMDCs were also incubated with anti-TLR2 or isotype control Abs without the addition of *L. johnsonii* N6.2 PLs.

**Figure 6.**
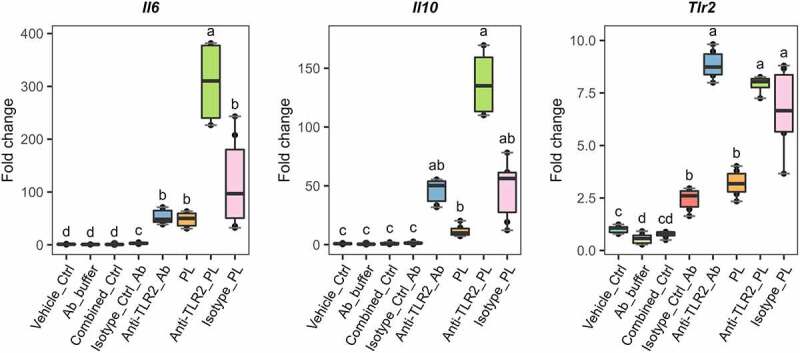
Antibody blocking of TLR2 on the surface of *L. johnsonii* N6.2 phospholipids-stimulated BMDCs. 7-day BMDCs were Ab-blocked with anti-TLR2 or isotype control Abs at 9 µg/mL for 1 h before addition of *L. johnsonii* N6.2 PLs at 5 µg/mL and further incubation for 6 h. Negative controls: vehicle control for PLs (Vehicle_ctrl), the Ab storage buffer (Ab_buffer) and both combined (Combined_ctrl). Ab controls: anti-TLR2_Ab and Isotype_Ctrl_Ab. Data was compared to Vehicle_Ctrl for statistical analysis. Data represent the values from three independent assays. Different letter labels denote statistically significant changes (p.value < 0.05).

Strikingly, the specific blocking of TLR2 with antibodies significantly enhanced the PL-mediated upregulation of *Il6* by six fold while *Il10* did not reach significance (Figure 6). The expression levels of *Tlr2* upon TLR2 Ab-blocking and PL stimulation were not significantly different, suggesting that the signaling induced by *L. johnsonii* N6.2 PLs does not greatly induce *Tlr2* expression. We also observed *Tlr2* induction upon stimulations with anti-TLR2 and isotype control Abs alone. We reasoned that the induction observed may be mediated by basal Fc receptor activity on the BMDCs. Indeed, to rule out basal Fc-receptor activity in the results depicted in Figure 6, we normalized gene expression changes to the isotype control treatment. The fold changes are depicted in Figure S9. This normalization of the data results in comparable statistically significant changes to the results presented in Figure 6. Overall, these results suggest that TLR2 signaling limits upregulation of pro-inflammatory *Il6* observed by the PL stimulation.

### Phospholipids from *L.* *johnsonii* N6.2 signal through the non-canonical NF-κB pathway

DCs are well equipped with a variety of pattern recognition receptors (PRRs) that recognize conserved microbial products generally known as pathogen-associated molecular patterns (PAMPs). DCs can sense a variety of PAMPs through TLRs and respond to the PAMP with a stimulus-specific gene expression program. A key feature of TLR signaling is the shared usage of NF-κB dimers downstream of TLR activation. Canonical NF-κB signaling in DCs regulates the pathogen-responsive gene expression patterns. As such, we utilized synthetic, cell permeable, NF-κB pathway inhibitors to determine whether the signaling induced by *L. johnsonii* N6.2 lipids was governed by the canonical NF-κB pathway. BMDCs were pre-incubated for 1 h with the IKKβ inhibitor IMD-0354 (IC_50_ = 250 nM, ab144823, Abcam) at three different concentrations (100, 250 and 500 nM) and with the IKKα inhibitor BMS-345541 (IC_50_ of 4 µM, ab144822, Abcam) at 5 µM. These inhibitors, and their concentrations, were selected to be able to discern canonical vs. non-canonical NF-κB signaling, as the non-canonical NF-κB pathway exclusively relies on the IKKα kinase alone.

After 1 h of incubation with the inhibitors, *L. johnsonii* N6.2 TLs or PLs were added to the cultures and incubated further for 6 more hours. As negative controls, cells were only incubated with the respective vehicle-controls (for *L. johnsonii* N6.2 lipids and synthetic inhibitors) combined as well as with the inhibitors alone for the duration of the assay. Cells were harvested and RNA was isolated for qRT-PCR gene expression analysis of *Il6, Il10* and *Tnf* (the latter as a prototypical, pro-inflammatory and canonical NF-κB-regulated gene).

Remarkably, inhibition of the IKKβ kinase alone (with IMD-0354), and hence, of canonical NF-κB signaling did not alter the expression levels of all three genes analyzed when BMDCs were treated with TLs (Figure S10). We observed a significant downregulation of *Il6* after PL stimulation only at the highest concentration of the inhibitor. However, there were not statistically significant differences between all three concentrations tested (Figure S10, left panel). Furthermore, the gene expression levels of *Tnf* were unchanged after IMD-0354 pre-treatment at all the concentrations evaluated in both TLs and PLs stimulations (Figure S10, right panel). These results suggest that canonical NF-κB signaling does not govern the gene expression patterns induced after *L. johnsonii* N6.2-lipid-stimulation of BMDCs.

**Figure 7.**
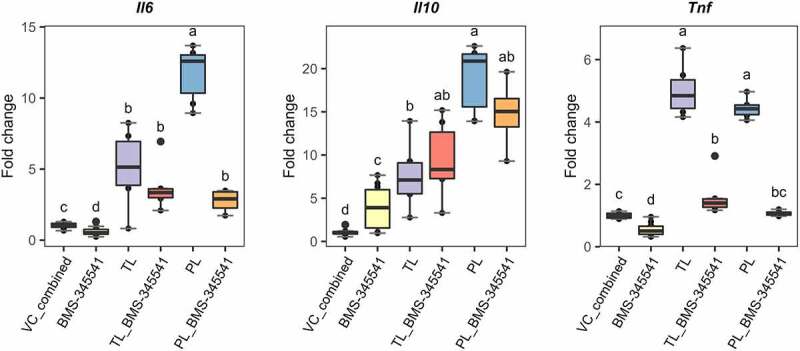
Blocking non-canonical NF-kB signaling in *Lactobacillus johnsonii* N6.2 lipid-stimulated BMDCs. 7-day BMDCs were pre incubated with the IKKα kinase inhibitor BMS-345541 at 5 µM for 1 h before addition of *L. johnsonii* N6.2 total lipids (TLs) or phospholipids (PLs) at 0.5 or 5 µg/mL respectively and further incubated for 6 h. Negative controls: vehicle control for *L. johnsonii* N6.2 lipids mixed with vehicle control for inhibitors (Vc_combined), NF- kB inhibitor alone. Data were obtained from three independent assays. Different letter labels denote statistically significant changes (p.value < 0.05).

Conversely, we observed a significant downregulation of *Tnf* to basal levels when the cells were pre-treated with BMS-345541 in both, TLs and PLs stimulations (Figure 7, left panel). A similar and significant downregulation was observed with the expression levels of *Il6* but only after stimulation of PLs (Figure 7, right panel). The results observed for this gene after stimulation with TLs did not reach significance and may reflect the fact that TLs represent a mixture of *L. johnsonii* N6.2-purified lipids. Finally, gene expression levels of *Il10* under BMS-345541 pre-treatment did not show significant differences for both, TL- and PL-stimulated BMDCs (Figure 7, middle panel); suggesting that non-canonical NF-κB signaling does not play a central role in the control of this gene.

Overall, the results described above suggest that the transcriptional signature observed after stimulation of BMDCs with phospholipids from *L. johnsonii* N6.2 is controlled in part, by non-canonical NF-κB signaling, including that of pro-inflammatory genes (as evidenced by *Il6* and *Tnf*).

## Discussion

The impact of dietary lipids on the immunological system plays a central role in many chronic diseases.^19,42^ As such, studies discussing the importance of dietary fats are overly abundant in the scientific literature.^43,44^ Immunological scape of pathogens linked to the modification of lipids by lipolytic enzymes in local inflammatory responses is also meticulously described.^45^ This vast amount of information contrasts with the scarcity of research papers describing the contribution of lipids synthesized by commensal bacteria on the tolerogenic immune response. The host has developed sophisticated mechanisms recognizing and accepting symbionts while rejecting pathogens. Commensals co-evolved with their host largely by promoting the development and education of a healthy immune system.^46^ In this context, commensals-synthesized lipids play a major role in the tolerogenic process. Indeed, a correlation between *A. muciniphila* genome features and unique *A. muciniphila* lipid species in the cecum has been recently found.^25^ Following this trend, recent studies have determined the immunoregulatory capacity of Gram-negative-commensal-derived lipids; like α-GalCers synthesized by *Bacteroides* spp.^19,22–24,27^ and more recently, of phospholipids and ornithine lipids from *A. muciniphila*. ^21,25^ However, the immunoregulatory capacity of Gram-positive-commensal-derived lipids has not yet motivated scientific attention.

We have previously shown that oral administration of *L. johnsonii* N6.2 induces a Th17 bias in the mesenteric lymph node of BBDP rats, consistent with immune stimulation exerted by the probiotic administration.^32^ The stimulation of BMDCs with *L. johnsonii* N6.2-purified lipids induced upregulation of *Il6, Il17f*, *Il1b, Il12b*, and *Il23a*. When *L. johnsonii* N6.2 was administered as a live-probiotic preparation, the strain induced transcriptional upregulation of *Il17a* and *Il23r*, along with induction of *Il6* and *Il23* in the mesenteric lymph nodes of the animals.^32^ The RNAseq-based analysis herein described could not identify induction of *Il17a* or *Il23r*, suggesting that upregulation of those genes may not be directly triggered by *L. johnsonii* N6.2-purified lipids. The cytokine receptors *Il6r* and *Il17ra* were downregulated (see Table S3), indicating that those cytokines might not act in an autocrine or in a trans-presenting manner.^47^ While IL-23 and IL-1β support a cellular differentiation toward the Th17 effector module,^48^ the expression of *Il17f* has been associated with nonpathogenic activity of TGF-β-polarized Th17 T cells.^49^

IL-10 is a potent anti-inflammatory and immunosuppressive cytokine known to repress functions of various innate and adaptive immune cells. Therefore, it suppresses inflammatory cytokine burst, prevents host damage, and maintains tissue integrity (reviewed in detail in).^50^ The induction of this cytokine in a pro-inflammatory context upon PRR stimulation mediates the resolution of inflammation after intestinal infections to restore homeostasis.^51–53^ We have previously described the upregulation of this cytokine after stimulation of human THP1 macrophages with *L. johnsonii* N6.2 nanovesicles.^17^ The results described herein suggest that the lipidic component of the nanovesicles may be responsible for such effects. Furthermore, the upregulation of anti-inflammatory cytokine *Il10* appears to be a specific response to *L. johnsonii* N6.2 phospholipids (Figure 4). This cytokine was not detected in culture supernatants of murine BMDCs and human monocytes stimulated with *A. muciniphila* phospholipids.^21^ The opposite effect was observed with TNFα. This cytokine was released by the monocytes after stimulation with *A. muciniphila* phospholipids, whereas it was slightly upregulated as detected by qRT-PCR and undetected by our RNAseq study. The immune-stimulating effects observed by *A. muciniphila* phospholipids were TLR2-dependent. The effects obtained with *L. johnsonii* N6.2 phospholipids were not dependent on TLR2 because they were enhanced after TLR2-Ab specific blocking (Figure 6).

Stimulation of BMDCs with *L. johnsonii* N6.2 lipids also induced the hematopoietic growth factors *Csf2* (GM-CSF) and *Csf3* (or granulocyte colony stimulating factor, G-CSF) along with several chemotaxis cytokines or chemokines: *Ccl2–5*, *Ccl20, Cxcl1–3, Cxcl6*, and *Cxcl11*. These molecules are important in cell migration as well as other biological processes, such as proliferation, survival, differentiation, and cytokine production. The chemokine groups CCL2–5 and CXCL1*-3*,6 mediate trafficking of innate immune cells, mainly of monocytes and granulocytes.^54^ CCL20 binds to CCR6 that is expressed on mature DCs, Th17, CD8+ T cells, Treg, and B cells.^55–58^ CXCL11 displays tolerogenic functions that restrain inflammatory autoimmune diseases. This chemokine induces Foxp3- regulatory T cells from both naïve T cells and disease-associated effector T cells in an autoimmune model.^59,60^ The induction pattern observed suggests that BMDCs stimulated with the probiotic lipids have the potential of mediating the migration of immune cells to the lymph nodes.

The obtained BMDCs displayed a cDC2-like transcriptional profile, evidenced by expression of *Itgax* (CD11c), *Itgam* (CD11b), and *Nfil3* observed after the stimulation. This DC subset can migrate from peripheral tissues to local LNs, where they present Ags to T cells and prime T-cell effector responses.^12^ *Clec10a* was downregulated, indicating that the BMDCs should be classified in the cDC2A subset.^39^ cDC2A were shown to possess anti-inflammatory and tissue-repair functions, as they secreted less pro-inflammatory cytokines IL-6 and TNF-α and polarizing activity toward IFNγ or IL-17A producing T cells.^39^

The purified lipids also induced the gene expression of *Tlr2, Tlr7, Nlrp3, Nod2*, and suppressed expression of *Tlr3* and *Tlr4* (see Table S3). A similar gene transcriptional pattern for *Tlr2, Tlr3, and Tlr7* was observed in macrophages responding to *L. johnsonii* N6.2 nanovesicles. That is, downregulation of *Tlr3* and upregulation of *Tlr2* and *Tlr7*. ^17^ NOD2 is an intracellular microbial sensor for both Gram-negative and Gram-positive bacteria while NLRP3 is a cytoplasmic protein that senses cellular stress and intracellular damage upon pathogen uptake. The synergistic activity of both NOD2 and NLRP3 has been shown to induce a particular profile of maturation markers (MHC-II, CD80, CD86); cytokines (IL-1α, IL-1β, IL-6, IL-2, IL-23p19 and IL-12p40); and chemokines (CXCL1 and 2) in murine BMDCs.^61^

We observed upregulation of costimulatory molecules *Cd86* and *Cd40*, downregulation of *Cd83* and upregulation of migratory markers *Marcks, Icam1, Ccr7*, and *Fscn1* along with moderate downregulation of CD62L (*Sell*). DCs migrate from non-lymphoid tissues into the draining LNs to present processed Ags to T cells. These migDCs display a mature state characterized by upregulated surface expression of MHC-I and -II along with CD86 and CD40. migDCs can transport both self- and exogenous Ags, and therefore exert tolerogenic or inflammatory T-cell responses (tol-migDC and inf-migDC, respectively).^41^ The surface expression levels of CD86 and CD40 along with MHC-II levels were not significantly increased. However, the migratory molecule ICAM-1 was highly increased at the surface of BMDC stimulated with both *L. johnsonii* N6.2-TLs and PLs. The phenotypic analysis of maturation markers supports the results transcriptionally observed (and described above). The downregulation of CD62L has been observed during T-cell activation and routinely reduced in these cells when transmigrating within LNs.^62^ These results suggest maturation of *L. johnsonii* N6.2-lipid-stimulated BMDC into a tol-migDC-like phenotype. This phenotype has important implications regarding DC function, as clonal anergy of T cells has been shown to be induced upon cognate stimulation in the presence of MCH-II and ICAM-1.^63^

Interestingly, we also observed upregulation of Jagged-1 (*Jag1)*. This Notch ligand has been shown to contribute to cell proliferation and Treg induction, sustaining the immunosuppressive environment (Reviewed in.^64^ Expression of Jagged-1 on mesenchymal stromal cells has been shown to induce expansion of existing Foxp3+ T reg cells.^65^ This effect was partially dependent on PD-L1 interactions on DCs.^66^ Indeed, an increased activation of peripheral memory Tregs after the washout period (p.value = 0.07) was observed after oral administration of live *L. johnsonii* N6.2 cells to healthy adults. While this trend did not reach significance, we hypothesize that *L. johnsonii* N6.2 lipids may play an important role in those observations.^29^

The NF-κB family of transcription factors is widely known for its central role in coordinating cellular responses to external stimuli. NF-κB dimers regulate the transcription of genes that control inflammation, immune cell development, cell survival, and proliferation (extensively reviewed in^67,68^). Activation of canonical NF-κB signaling depends on the kinase IKKβ, whereas non-canonical signaling entirely relies on IKKα alone. The classical (canonical) NF-κB signaling is a hallmark of inflammatory challenge in both immune and nonimmune cells. It is activated by a variety of stimuli, including binding of PAMPs, damage-associated molecular patterns (DAMPs) and pro-inflammatory cytokines to their cognate receptors. Hence, dysregulations of canonical NF-κB signaling are contributed to the pathogenesis of various inflammatory diseases.^69,70^ Given the importance of the classical pathway in an immunological context, we assessed whether *L. johnsonii* N6.2-purified lipids trigger its activation in BMDCs. Strikingly, pre-treatment of BMDCs with the IKKβ inhibitor IMD-0354 did not significantly change the gene expression levels of *Il6, Il10* and *Tnf* induced by *L. johnsonii* N6.2 TLs and PLs at all the concentrations tested (see Figure S10). In contrast, highly selective inhibition of IKKα (by BMS-345541) led to a significant downregulation of *Il6* and *Tnf* to basal levels (Figure 7).

Recent studies have demonstrated that the expression control of NF-κB-targeted genes is not only stimulus-specific but also cell type-specific.^71,72^ Moreover, crosstalk with non-canonical NF-κB signaling may either extend or restrain the duration of canonical NF-κB activation in a cell type-specific manner.^67,73^ In DCs, non-canonical NF-κB activity restrains maturation under steady-state conditions, but upon TLR ligation, canonical signaling is activated and regulates the expression of inflammatory gene programs and DC-surface activation markers.^74^ While different PAMPs are known to activate canonical NF-κB signaling downstream of TLR-ligation,^72^ little is known about the involvement of PAMPs, and particularly of commensal-derived bioactive lipids in stimulating DCs through a non-canonical NF-κB-manner. We observed that the gene expression of *Tnf* is specifically controlled by the non-canonical NF-κB pathway. This gene has been previously demonstrated to show a codependency either on canonical or non-canonical NF-κB transcription factors in DCs.^74^ Moreover, Baratin et al.^75^ demonstrated that homeostatic NF-κB signaling in DCs is required to maintain tissue immune tolerance, and that steady-state migDCs displayed a different NF-κB-regulated network of genes that support DC immune homeostasis as opposed to inflammatory conditions. Our NF-κB-inhibition experiments suggest that *L. johnsonii* N6.2-purified lipids activate a tol-mig-like gene expression program that is mediated in part by non-canonical NF-κB signaling. This response is hence highly specific to the stimulus presented and to the cells stimulated.

Cell-based assays identified the phospholipid (PL) fraction as the immune-stimulating, bioactive fraction. Phospholipids (specifically glycerophospholipids, GP) represent 28% of *L. johnsonii* N6.2-purified lipids (Figure 1a). Among those GPs, 90% represented diacylglycerophosphoglycerols or phosphatidylglycerols (PGs) (see Figure S7b). The major phospholipids in *Lactobacillus* spp. are cardiolipins (CLs), GPs and dihexaosyl diacylglycerols (DH-DGs).^16^ Interestingly, GPs enriched in *L. johnsonii* N6.2 nanovesicles include PGs such as PG(16:0_18:1), PG(18:1_18:1), PG(17:1_20:2), PG(37:3) and PG(36:2).^16^ The anionic pulmonary surfactant PG(16:0_18:1) or palmitoyl-oleoyl-PG (POPG) and the di-oleoyl PG (DOPG) or PG(18:1_18:1) have been shown to inhibit TLR2 and TLR4 activation in murine macrophages upon stimulation with either the TLR ligands LPS (TLR4), Pam3Cys (TLR2/1), MALP-2 (TLR2/6), lipoprotein (TLR2) or *Mycoplasma pneumoniae* membranes for POPG or with the antimicrobial peptide S100A9 for DOPG.^76,77^ Furthermore, the anti-inflammatory effects of DOPG on S100A9-stimulated macrophages are driven by inhibition of p65 (a canonical NF-κB TF) phosphorylation and translocation to the nucleus.^77^ POPG also decreases influenza A viral-induced inflammation when administered to mice intranasally.^78^ Moreover, drug delivery systems based on anti-inflammatory PGs are also being explored. As such, phosphatidylserine (PS) and PG as DOPS- or DOPG-formulated mixed micelles and liposomes were shown to suppress TNFα production from mouse peritoneal macrophages stimulated with IFNγ or LPS.^79^

Overall, our analysis reveals that BMDCs stimulated with *L. johnsonii* N6.2-purified lipids share similar transcriptional signature to that of steady state migDCs, including upregulation of maturation-mig related genes *Cd86, Cd40, Ccr7, Marcks, Icam1*, and immunoregulatory genes that include *Itgb8, Nfkbiz, Jag1, Hcar2, Adora2a, IL2ra, Arg1, Cd274*, and *Cd200*. This gene expression program suggests the partial involvement of by TLR2-signaling and the non-canonical NF-κB pathway in a context- and cell type-specific manner. These BMDCs present a tol-migDC-like phenotype as determined by surface expression of maturation and migratory markers. Our results herein provide insights into how Gram-positive-commensal-derived bioactive lipids may regulate and educate cellular immunity in the gut and further suggest that *L. johnsonii* N6.2 lipid stimulated-BMDCs have the potential of dampening immune responses and induce/promote regulatory T cell responses, a subject of further investigation.

## Experimental procedures

### Bacterial cultures

*L. johnsonii* N6.2 was cultivated under anaerobic, static conditions in de Man, Rogosa, Sharpe (MRS) media. For EPS isolation, MRS media was subjected to ultracentrifugation (Beckman) at 175 000 × *g* for 2 h at 4°C followed by filter-sterilization (0.2 µm), to remove any EPS from the yeast extract present as part of the media formulation. *L. johnsonii* N6.2 was inoculated in MRS at 1% (v/v) and incubated at 37°C for 24 h for total lipid extraction, or for 48 h for EPS isolation. *E. coli* MG1655 and *B. subtilis* 168 were cultured in Luria-Bertani (LB) broth at 37°C for 24 h under shaking conditions.

### EPS isolation

1 L cultures of *L. johnsonii* N6.2 grown for 48 h were harvested by centrifugation at 15,900 × *g* for 20 min at 4°C. The culture supernatant was collected as well as supernatants generated following the subsequent cell pellet washes. Cell pellet was resuspended and washed twice with PBS pH 7.2 and once with Milli-Q water. The washed biomass was frozen at −80°C overnight and freeze-dried (Labconco) for 24 h. The cell culture supernatant and wash supernatants were combined. EPS was extracted from both the cell biomass and the culture supernatants as separated procedures following the methods by Górska et al.^80^ and Horn et al.,^81^ respectively. In brief, freeze-dried biomass was treated with 10% TCA for 2.5 h at room temperature (RT) followed by centrifugation at 14 500 × *g*, 20 min. The EPS contained in the supernatant was then precipitated with 5× V of cold 96% ethanol overnight (kept at 4°C) and collected by centrifugation at 23 500 × *g*, 50 min. The pellet was resuspended in water (with gentle heating at 37°C if needed), dialyzed (in water) for 48 h, lyophilized and stored at 4°C.

EPS from the culture supernatants was precipitated by adding an equal volume of ethanol and incubated at 37°C overnight. Precipitated EPS was collected by centrifuging at 14 500 × *g*, 20 min. EPS in the pellet was resuspended in water (with gentle heating) and precipitated with 2× V of chilled (−20°C) ethanol. The EPS was recovered by centrifugation at 14 500 × *g*, 20 min. Precipitated EPS was resuspended in water and dialyzed for 72 h. Culture supernatant EPS was further purified by dissolving in 10% TCA overnight. Precipitated protein was removed by centrifugation at 10,000 × *g* for 15 min, 4°C. The supernatant pH was adjusted to 7 with 1 M NaOH and EPS was precipitated again with 2× Volume of chilled ethanol, centrifuged, and resuspended in water prior to its lyophilization and storage at 4°C.

EPS from bacterial cells and culture supernatants were further cleaned by resuspending in buffer (50 mM Tris-HCl pH 7.5, 10 mM MgCl_2_) and treating with DNase I (2 units/100 µL) and RNase H (5 units/100 µL) (New England Biolabs) at 37°C for 6 h followed by a proteinase K (Fisher scientific) treatment (100 ug/mL) overnight (37°C). After all digestions, EPS was re-purified as described above for culture supernatants, lyophilized, and stored at 4°C.

### Total lipid extraction and fractionation

Bacterial cells from 24 h-*L. johnsonii* N6.2, *E. coli* MG1655, or *B. subtilis* 168 cultures were harvested by centrifugation at 15,900 × g for 20 min at 4°C. Cells were washed twice with 1% (w/v) NaCl, frozen at − 80°C overnight and freeze-dried (Labconco) for 24 h. Total lipids from the bacterial strains were extracted using a modified Bligh and Dyer method.^82^ In short, one gram of freeze-dried cells was added into a clean glass separatory funnel. A total of 114 mL of solvents were added in sequence to achieve a final cloroform:methanol:water ratio of 1:2:0.8 (v/v/v). All solvents used were ACS or HPLC grade. The mixture was allowed to stand for 18 h with occasional shaking and subsequent phase separation was achieved by adding chloroform and water to reach a chloroform:methanol:water ratio of 1:1:0.9 (v/v/v). The lower chloroform phase was collected. The majority of the solvent was evaporated in a rotavapor (Buchi) at 474 mbar and a water bath of 40°C. Remaining solvent was evaporated under a nitrogen stream and dried lipid extractions were sealed in glass tubes and stored at −80°C.

Total lipids (TL) from *L. johnsonii* N6.2 were fractionated by column chromatography using a modified Frostegård method.^40^ In brief, pooled total lipids extracted from a large-scale culture (14 g of freeze-dried biomass) were resuspended and combined in chloroform:acetic acid (100:1 v/v); and loaded to a column (3.2 × 45.7 cm) of oven-activated (125°C) silica gel 60 (0.063–0.200 mm, Millipore) conditioned with the same solvent mixture. A simple lipid fraction (SL, including fatty acids) was then eluted with chloroform:acetic acid (100:1 v/v, 1200 mL), followed by a glycolipid (GL) fraction eluted with acetone (2400 mL) and a phospholipid (PL) fraction eluted with methanol (1200 mL). The solvents were evaporated as indicated above. Individual lipid fractions were aliquoted as needed in glass tubes, and remaining solvent was evaporated under a stream of nitrogen. Dried lipid amounts obtained were measured gravimetrically. Lipid fractions were stored at −80°C until further use.

### Untargeted qualitative lipidomics

Qualitative LC-MS/MS Analysis was performed to follow the lipid fractionation process. The service was provided by the Mass Spectrometry Research and Education Center, Department of Chemistry, University of Florida, Gainesville, FL, United States. Two biological aliquots of TL and lipid fractions (SL, GL and PL) were used for this analysis. Dried lipid samples were reconstituted in chloroform (TL), 1:1 DMSO:ethanol (SL and GL), and 1:1 chloroform:ethanol (PL) and diluted further in ethanol. A portion of the dilute sample was transferred to an autosample vial for LC-MS/MS analysis. The samples were analyzed by reversed phase (Acclaim PepMap C18) under a 70 min gradient. Mobile phases of isopropanol, acetonitrile, and water, both containing ammonium formate and formic acid was used for positive polarity LC-MS/MS and negative polarity LC-MS/MS. Eluent was analyzed on a QTOF-MS with data-dependent MSMS programmed for fragmentation of singly charged ions between m/z 500–1500 for positive mode and ions between m/z 250–1500 for negative mode. The MS(MS) data was then searched using lipidomic libraries in Metaboscape and SimLipid.

### Animals

Sprague-Dawley (SD) rats were purchased from Charles River Laboratories (USA). Animals were housed under a 12-h light:dark cycle and controlled climate as prescribed by the Association for Assessment and Accreditation of Laboratory Animal Care. All animals were fed standard rodent chow and water *ad libitum*. Experiments were performed with 8–12 weeks old rats. All animal procedures were approved by the University of Florida Animal Care and Use Committee with protocol #202011186.

### Primary cell cultures

Bone marrow progenitors were isolated from SD rats (8–12 weeks old) by flushing femurs with Hank’s Balanced Salt Solution (HBSS). Bone marrow progenitors were resuspended in RPMI-1640 supplemented with 10% heat-inactivated fetal bovine serum (FBS, Sigma), 2 mM L-glutamine (as GlutaMAX^TM^ supplement), 50 µM 2-mercaptoethanol (Sigma), 100 units/mL of penicillin, 100 µg/mL streptomycin (complete medium, all components from Gibco unless otherwise stated). Complete RPMI-1640 medium was supplemented with 20 ng/mL of rat GM-CSF and 200 ng/mL of human FLT3-L (both from R&D Systems) to generate bone marrow-derived dendritic cells (BMDCs) (complete BMDC medium). BMDCs were cultured in non-treated *T*-75 flasks (Thermo Scientific) for 7 days at 37°C and 5% CO_2_ with medium changes every 2–3 days. On day 7, the loosely adherent and nonadherent cells were harvested for the cell-based assays. Flow cytometry with anti-rat CD11b/c antibody (Clone OX-42, BioLegend) was used to confirm the purity of 7-d non-adherent BMDCs. Figure S11 displays the gating strategy followed for flow cytometry analysis. Purity was routinely > 90%.

### Lipid stimulation experiments

For our RNAseq-based experiment, TLs were resuspended in PBS pH 7.2. For our bioassay-guided fractionation experiments, TL, SL and GL were resuspended in DMSO:ethanol (1:1 v/v). PL was resuspended in DMSO:ethanol:PBS (1:1:2 v/v/v). Lipid suspensions were vortexed at max speed and heated up to 40°C for 10 min in a heat block to aid in resuspension. This process was repeated one more time before further dilutions. Working lipid suspensions were prepared by diluting 1:4 in PBS. The solvent mixture DMSO:ethanol:PBS (1:1:6 v/v) constituted the vehicle solution as it represented the mixture with the greater amount of solvents. Working suspensions were further diluted in the vehicle if needed prior to addition to complete BMDC medium. Equal volumes of working lipid suspensions were added to the medium to achieve the concentrations assayed. Synthetic α-GalCer (KRN700) was purchased from Adipogen, dissolved in DMSO following manufacturer’s recommendations, diluted 1:10 in PBS pH 7.2 and added to the cells at 100 ng/mL. 7-d nonadherent BMDCs were added to a 6-well non-treated plate (Thermo Scientific) at 10^6^ cells per well in 3 mL of complete BMDC medium supplemented with either the lipid treatments or the vehicle control. Cells were incubated at 37°C and 5% CO_2_ for 6 h and harvested for RNA isolation and immunostaining.

### MTT assay

7-d nonadherent BMDCs were added to a 96-well non-treated plate at 10^4^ cells per well and 200 µL of complete BMDC medium supplemented with either the lipid treatments at evaluated concentrations or the vehicle control. Cells without any treatment were also assayed as a positive control. Complete BMDC medium alone was added as a blank control. Cells were incubated at 37°C and 5% CO_2_ for 7 h. After the incubation period, 20 µL of MTT (as a 5 mg/mL solution in PBS) was added to each well and further incubated for 2 more hours. Subsequently, the plate was centrifuged to pellet down the cells at 300 × *g* for 5 min, the culture medium removed and the MTT crystals were dissolved with 200 uL of acidified isopropanol (prepared by adding 50 mL of 2 M HCl to 2.5 L of isopropanol). The generated amount of blue formazan was determined spectrophotometrically at 570 nm.

### RNAseq analysis

After isolation of total RNA, library construction and sequencing were performed by Novogene, (Novogene Co., Davis, CA, USA). A total amount of 1 µg RNA per sample was used as input material for the RNA sample preparations. Sequencing libraries were generated using NEBNext® Ultra TM RNA Library Prep Kit for Illumina® (NEB) following manufacturer’s recommendations and index codes were added to attribute sequences to each sample. Briefly, mRNA was purified from total RNA using poly-T oligo-attached magnetic beads. Fragmentation was carried out using divalent cations under elevated temperature in NEBNext First Strand Synthesis Reaction Buffer (5X) or by using sonication with Diagenode bioruptor Pico for breaking RNA strands. First strand cDNA was synthesized using random hexamer primer and M-MuLV Reverse Transcriptase (RNase H). Second strand cDNA synthesis was subsequently performed using DNA Polymerase I and RNase H. Remaining overhangs were converted into blunt ends via exonuclease/polymerase activities. After adenylation of 3’ ends of DNA fragments, NEBNext Adaptor with hairpin loop structure were ligated to prepare for hybridization. In order to select cDNA fragments of preferentially 150 ~ 200 bp in length, the library fragments were purified with AMPure XP system (Beckman Coulter). Then 3 µl USER Enzyme (NEB) was used with size-selected, adaptor-ligated cDNA at 37°C for 15 min followed by 5 min at 95°C before PCR. Then PCR was performed with Phusion High-Fidelity DNA polymerase, Universal PCR primers and Index (X) Primer. At last, PCR products were purified (AMPure XP system) and library quality was assessed on the Agilent Bioanalyzer 2100 system. The clustering of the index-coded samples was performed on a cBot Cluster Generation System using PE Cluster Kit cBot-HS (Illumina) according to the manufacturer’s instructions. After cluster generation, the library preparations were sequenced on an Illumina platform and paired-end reads were generated. Raw data (raw reads) of FASTQ format were firstly processed through fastp to generate clean reads.^83^ Paired-end clean reads were mapped to the reference genome using HISAT2 software.^84^ Novogene Co., Ltd Quantification FeatureCounts^85^ was used to count the read numbers mapped of each gene.

Raw counts normalization and filtration, along with differential expression analysis were performed with RStudio version 2022.7.1.554 as follows: RNAseq length and GC content biases correction was performed with the EDAseq package v2.30.0^86^ for both, within and between lane normalizations. Outlier detection was performed with the iterative leave-one-out (*iLOO*) approach, described by George et al.^87^, and filtration of low-count data was performed with the NOISeq package v2.40.0.^88,89^ Exploratory analyses were performed with the graphical tools of NOISeq and ComplexHeatmap v2.12.1.^90,91^ Pairwise comparisons were performed with NOISeqBIO from the package NOISeq. The probability of differential expression applying NOISeqBIO is 1-FDR, where FDR is considered as an adjusted p-value and no further adjusting was performed. Significant differentially expressed genes (DEGs) were defined as those DEGs with a p.adjust < 0.01 and an absolute log2FC value greater than 0.3. Gene ontology (GO) enrichment analysis was performed with the package goseq v1.48.0^92^ and over-represented p-values were adjusted with the Benjamini–Hochberg method.

### RT-qPCR analyses

Total RNA was isolated with the RNeasy mini kit (Qiagen) according to the manufacturer’s instructions. DNA was digested with the TURBO DNA-*free*^TM^ kit (Invitrogen). cDNA was produced with the iScript^TM^ cDNA synthesis kit (Bio-Rad) and qPCR assays were performed using the PowerUp™ SYBR™ Green Master Mix (Applied Biosystems) in a QuantStudio 6 machine (Applied Biosystems). Glyceraldehyde-3-phosphate dehydrogenase (GAPDH) was used as an internal control in each experiment. All primers were designed to specifically bind rat cDNA. The sequence of primer used are shown in Table S5.

### TLR2 blocking experiment

TLR2 on the surface of 7-d BMDCs was blocked by pre-incubating the cells with 9 µg/mL of recombinant monoclonal anti-rat TLR2 antibody (ab209217, Abcam) or isotype control (ab172730, Abcam) for 1 h prior to the addition of 5 µg/mL of *L. johnsonii* N6.2 PLs (as described above) and further incubation for 6 h. The antibody storage buffer (59% PBS pH 7.2, 40% glycerol, 0.05% BSA, 0.01% sodium azide) was prepared in-house and added at equal volumes as a negative control along with the vehicle control for the lipids. Cells were also treated with the Abs alone throughout the duration of the assay. 3∙10^5^ BMDCs per well were assayed in 1 mL of complete BMDC medium in a 12-well non-treated plate (Thermo Scientific).

### Inhibition of NF-κB signaling

The NF-κB signaling pathway was inhibited by 1 h pre-treatment with the IKKβ inhibitor IMD-0354 (IC_50_ = 250 nM, ab144823, Abcam) at three different concentrations (100, 250, and 500 nM) and with the IKKβ and IKKα inhibitor BMS-345541 (IC_50_ of 0.3 and 4 µM, respectively, ab144822, Abcam) at 5 µM. Stock solutions of the inhibitors at 25 mM were prepared in DMSO following manufacturer’s recommendations, further diluted in PBS pH 7.2 and added to complete BMDC medium as PBS-diluted solutions to achieve the concentrations analyzed. The vehicle control for the inhibitors constituted the least diluted DMSO:PBS solution (1:2). As a negative control, both vehicle controls (for lipids and inhibitors) were combined and added to the cells at equal volumes as their tested counterparts. The inhibitors alone were also assessed throughout the duration of the assay. *L. johnsonii* N6.2 lipids were added as TLs or PLs at 0.5 or 5 µg/mL and cells were stimulated for 6 additional hours. 3∙10^5^ 7-d BMDCs per well were assayed in 1 mL of complete BMDC medium in a 12-well non-treated plate (Thermo Scientific).

### Flow cytometry

BMDCs phenotype was characterized with a panel of cell surface, fluorescent dye-conjugated/biotinylated mouse anti-rat antibodies (Abs). All centrifugation steps were performed at 300 × g, 5 min. An Ab cocktail was prepared by combining 50 µL of BD Horizon™ Brilliant Stain Buffer (BD Biosciences) with PerCP-OX-6 Ab (anti-MHC-II, BD Biosciences), PE/Cy7-OX-42 Ab (anti-CD11b/c, BioLegend), biotin-3H5 Ab (anti-CD80, Thermo Fisher Scientific), FITC-24F Ab (anti-CD86, BioLegend), APC-HM40-3 Ab (anti-CD40, Thermo Fisher Scientific), BV711-1A29 Ab (anti-ICAM-1, BD Biosciences) and BV421-WTH1 Ab (anti-CD1d, BD Biosciences). Cells were harvested by centrifugation and washed twice with PBS prior to viability staining with the eFluor^TM^ 780 fixable viability dye (FVD) (Invitrogen) for 30 min at 4°C. Cells were washed twice with staining buffer (BioLegend) and blocked with mouse anti-rat CD32 Ab for 10 min. The Ab cocktail was then added, and cells were incubated for an additional 30 min at room temperature (RT). Cells were washed twice with staining buffer and fixed with the Cyto-Fast™ Fix/Perm Buffer Set (BioLegend) according to the manufacturer’s recommendations. Flow cytometry was performed with an Aurora 5 (Cytek) instrument and the data were analyzed with the FCS Express 7 Flow software.

### Statistical analyses

RStudio was used to perform statistical analysis. To identify genes with statistically significant differences between treatments, a one-way ANOVA followed by a Tukey post-hoc test was performed. Median fluorescence intensity (MFI) values obtained from the immunophenotyping experiments were normalized to their respective vehicle control, converted into log2 values, and analyzed by a one-way ANOVA and a Tukey test as described for qRT-PCR data. Statistical significance was defined as p.value < 0.05.

## Supplementary Material

Supplemental Material

## Data Availability

RNAseq data is deposited at the Gene Expression Omnibus (GEO; accession number: GSE221588). Lipidomics data generated in this study is provided in this manuscript as Table S2. Datasets for Table S2 and Table S3 have been uploaded to the Zenodo repository and can be found following the DOIs: 10.5281/zenodo.8044437 and 10.5281/zenodo.8044444, respectively.
