# Supplementary material for: Lactobacillus johnsonii N6.2 phospholipids induce immature-like dendritic cells with a migratory-regulatory-like transcriptional signature
Source: Gut Microbes. 2023 Sep 7;15(2):2252447. doi: 10.1080/19490976.2023.2252447 (PMC10486300; doi:10.1080/19490976.2023.2252447)
Supplement: Supplemental Material [file KGMI_A_2252447_SM2693.zip › Supplementary material/Supplemental material_revised_v2.docx]

*Lactobacillus johnsonii* N6.2 phospholipids induce immature-like dendritic cells with a migratory-regulatory-like transcriptional signature

Alexandra E. Cuaycal^1^, Leandro Dias Teixeira^1^, Graciela L. Lorca^1^ and Claudio F. Gonzalez^1*^

# Supporting information

**Table S1. *Lactobacillus johnsonii* N6.2 total lipid profiling by qualitative lipidomic LC- MS/MS analysis.** The MS-2 annotations (precursor mass- and spectral-matched) detected are presented. RT = retention time. m/z = mass-to-charge ratio. Category: GCL* = Glycerolipid; GP

= glycerophospholipid; FA = fatty acyl. Table is attached as a word file. *: In the table, the abbreviation GCL for glycerolipids was selected to avoid confusion with GL (denotation for the glycolipid fraction obtained after lipid fractionation of *L. johnsonii* N6.2 total lipids).

**Table S2. *Lactobacillus johnsonii* N6.2 total lipid and fractionated lipids profiling by qualitative lipidomic LC-MS/MS analysis.** Table presents both MS-1 (precursor mass- matched) and MS-2 (precursor mass- and spectral-matched) annotations. The values in the table represent the peak area. The column labels, RT: retention time; m/z: mass-to-charge ratio; SL (simple lipids), GL (glycolipids), and PL (phospholipids) represent lipid fractions and TL: total lipids. Table is attached as an xlsx file.


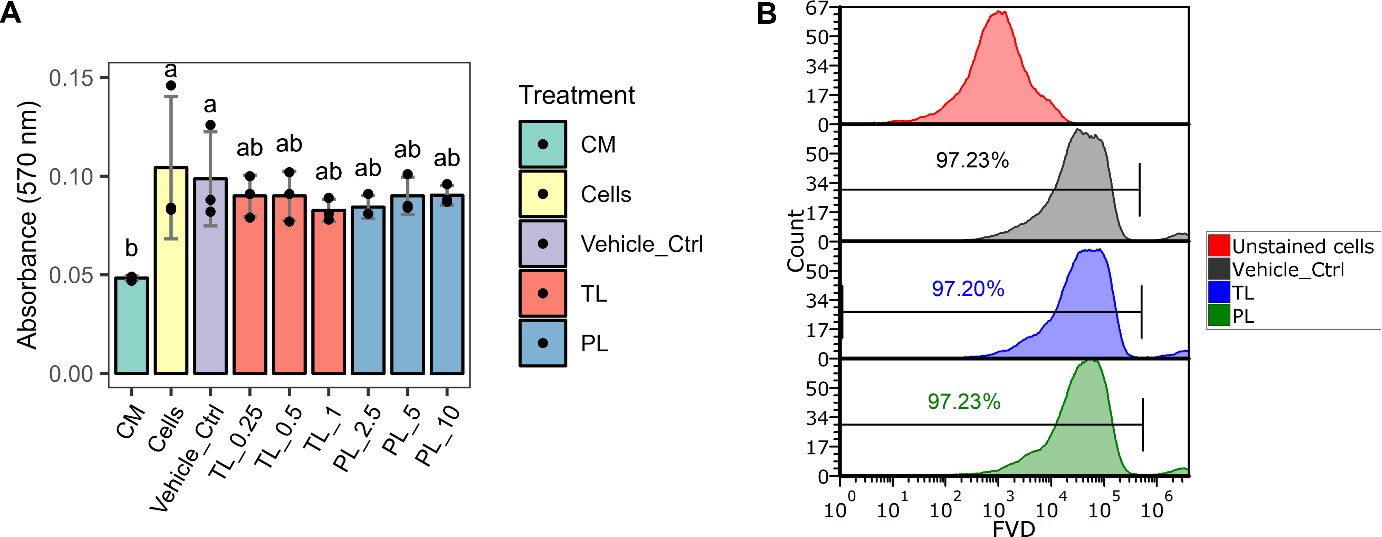


**Figure S1. Assessment of viability of BMDCs after treatment with purified lipids.** A. Metabolic state of BMDCs was assessed by the MTT assay. Total lipids (TL) were evaluated at 0.25, 0.5 and 1 µg/mL. The phospholipid fraction (PL) was evaluated at 2.5, 5 and 10 µg/mL. CM = complete culture medium**.** B. Cell viability of BMDCs as determined by flow cytometry with the eFluor^TM^ 780 fixable viability dye (FVD) (Invitrogen). Data is representative of three independent assays. Different letter labels denote statistically significant changes (p.value < 0.05).


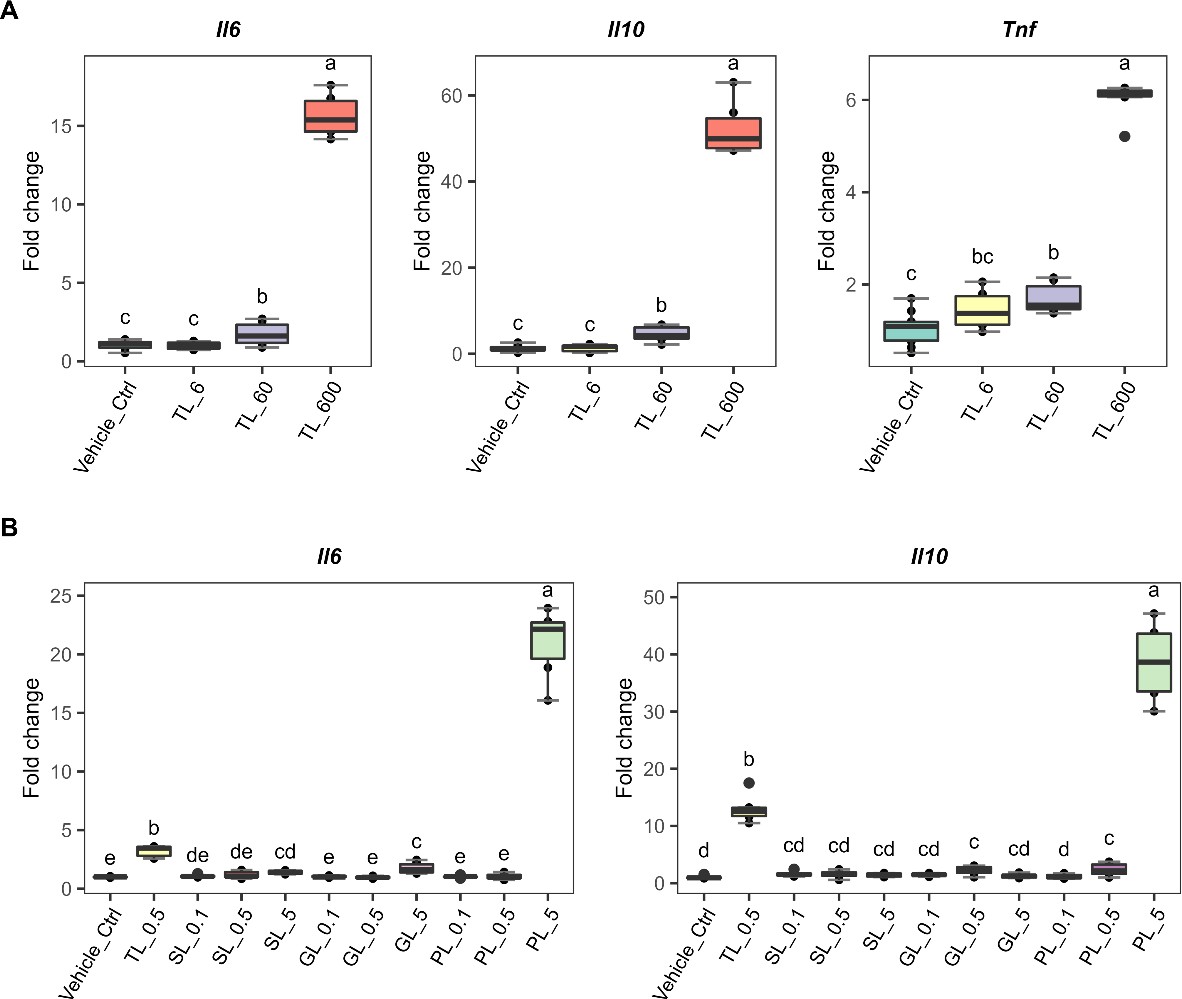


**Figure S2. Stimulatory capability of *L. johnsonii* N6.2 purified lipids as determined by qRT- PCR of *Il6*, *Il10* (A & B) and *Tnf* (A).** A. Total lipids were assayed in three different concentrations: 6, 60 and 600 ng/mL. B. The purified lipid fractions: simple lipids (SL), glycolipids (GL) and phospholipids (PL) were assayed at 0.1, 0.5 and 5 µg/mL. TLs at 0.5 µg/mL were included as a positive control. In both experiments, the vehicle control was included as a negative control. Data represents three independent assays. Different letter labels denote statistically significant changes (p.value < 0.05).


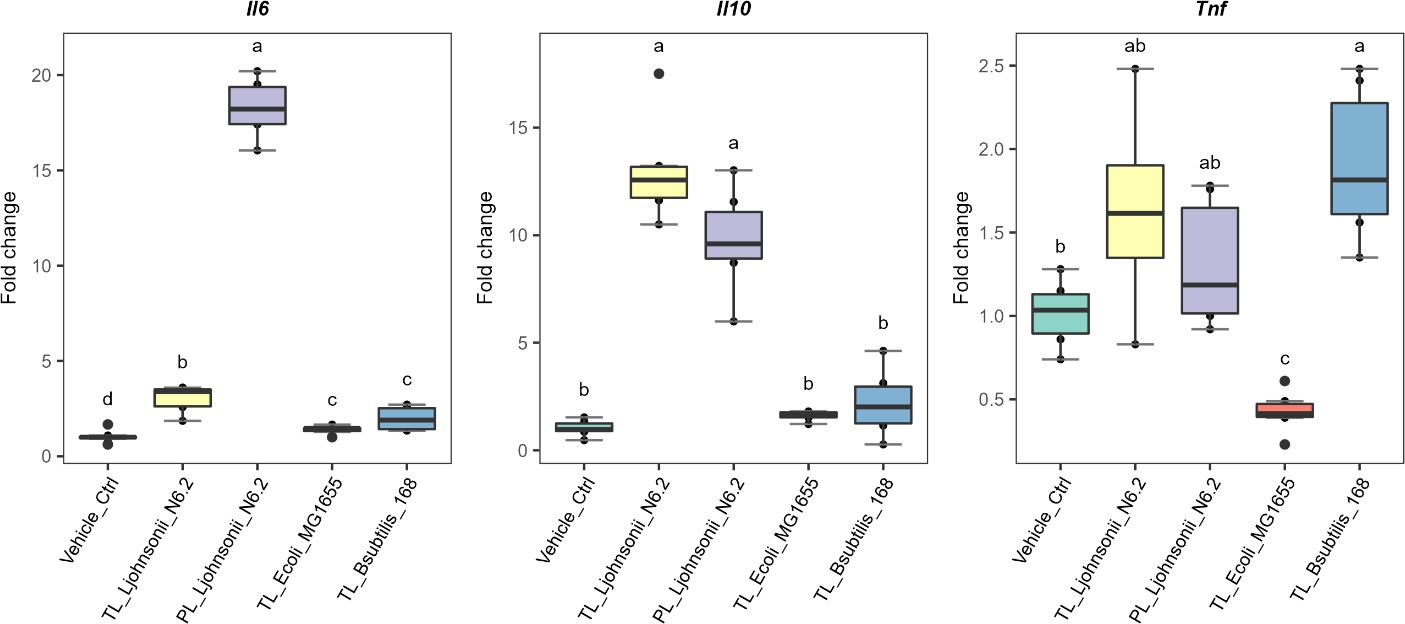


**Figure S3. Stimulatory capability of *L. johnsonii* N6.2, *E. coli* MG1655 and *B. subtilis* 168 purified lipids as determined by qRT-PCR of *Il6*, *Il10* and *Tnf*.** Total lipids for all bacteria were assayed at 0.5 µg/mL. PLs from *L. johnsonii* N6.2 were assayed at 5 µg/mL. The vehicle control was included as a negative control. BMDCs were stimulated for 6 h. Data represents three independent assays. Different letter labels denote statistically significant changes (p value < 0.05).

**Table S3. List of significantly differentially expressed genes (p.ajust < 0.01, |log2FC| > 0.3).** DEGs were obtained by pair-wise differential expression analysis of *Lactobacillus johnsonii* N6.2 total lipid stimulation vs. the vehicle control. Table is attached as an xlsx file.


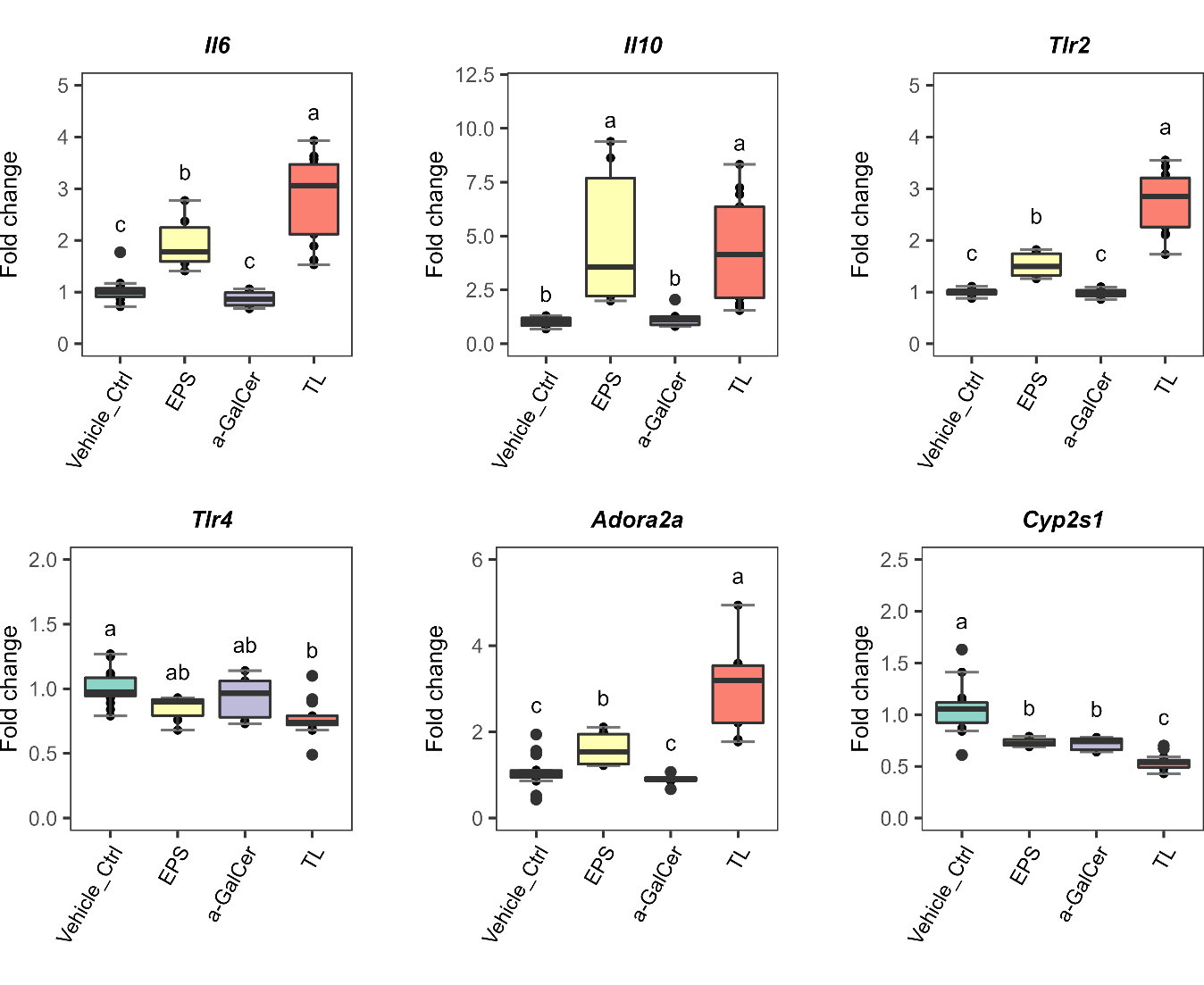


**Figure S4. Gene expression of selected response-markers.** BMDCs were stimulated with *L johnsonii* N6.2 total lipids (TL) or exopolysaccharides (EPS) at 500 ng/mL or α- (a-)GalCer at 100 ng/mL versus the vehicle control. Data were obtained from three independent assays.

Different letter labels denote statistically significant changes (p.value < 0.05).

**Table S4. Gene ontology (GO) enrichment analysis.** Table presents all significantly enriched GO terms (p.adjust < 0.01) obtained after analysis with DEGs with an absolute log2FC ≥ 1.

Table is attached as an xlsx file.


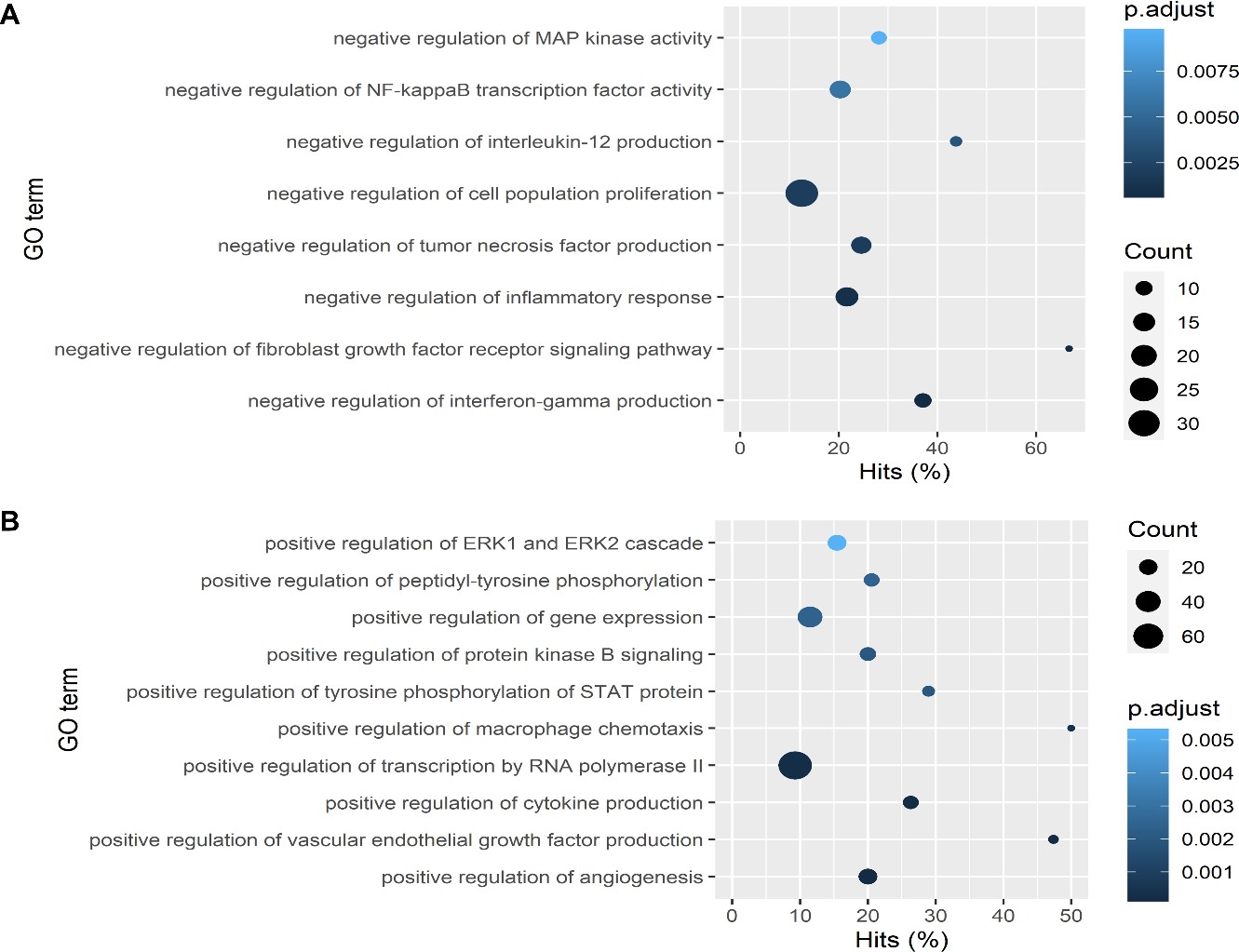


**Figure S5. Negatively and positively regulated GO terms after BMDC stimulation with *Lactobacillus johnsonii* N6.2 total lipids.** GO enrichment analysis was performed with the DEGs with an absolute log2FC ≥ 1. A: negatively regulated GO terms. B: top 10 positively regulated GO terms.


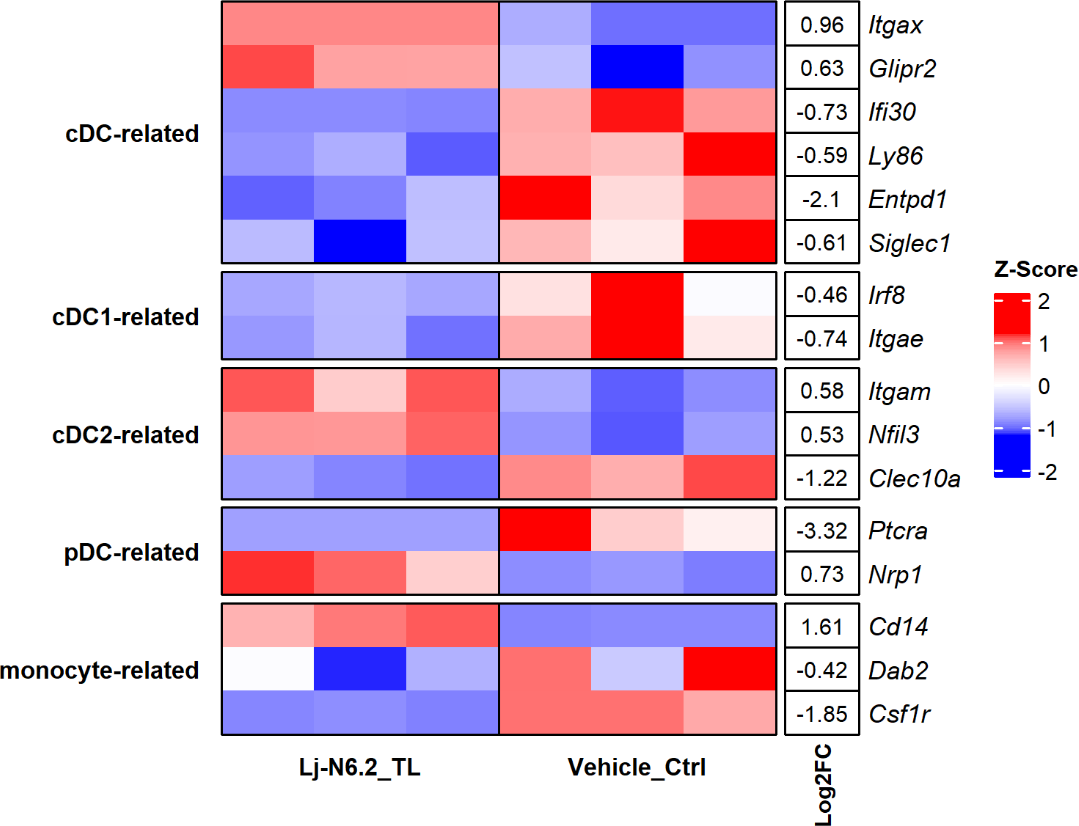


**Figure S6. Heatmap representation of differential gene expression of classical and plasmacytoid DC subset-related genes (p.ajust < 0.01).** BMDCs were stimulated with *Lactobacillus johnsonii* N6.2 total lipids or the vehicle control for 6h. Data represent three independent assays.


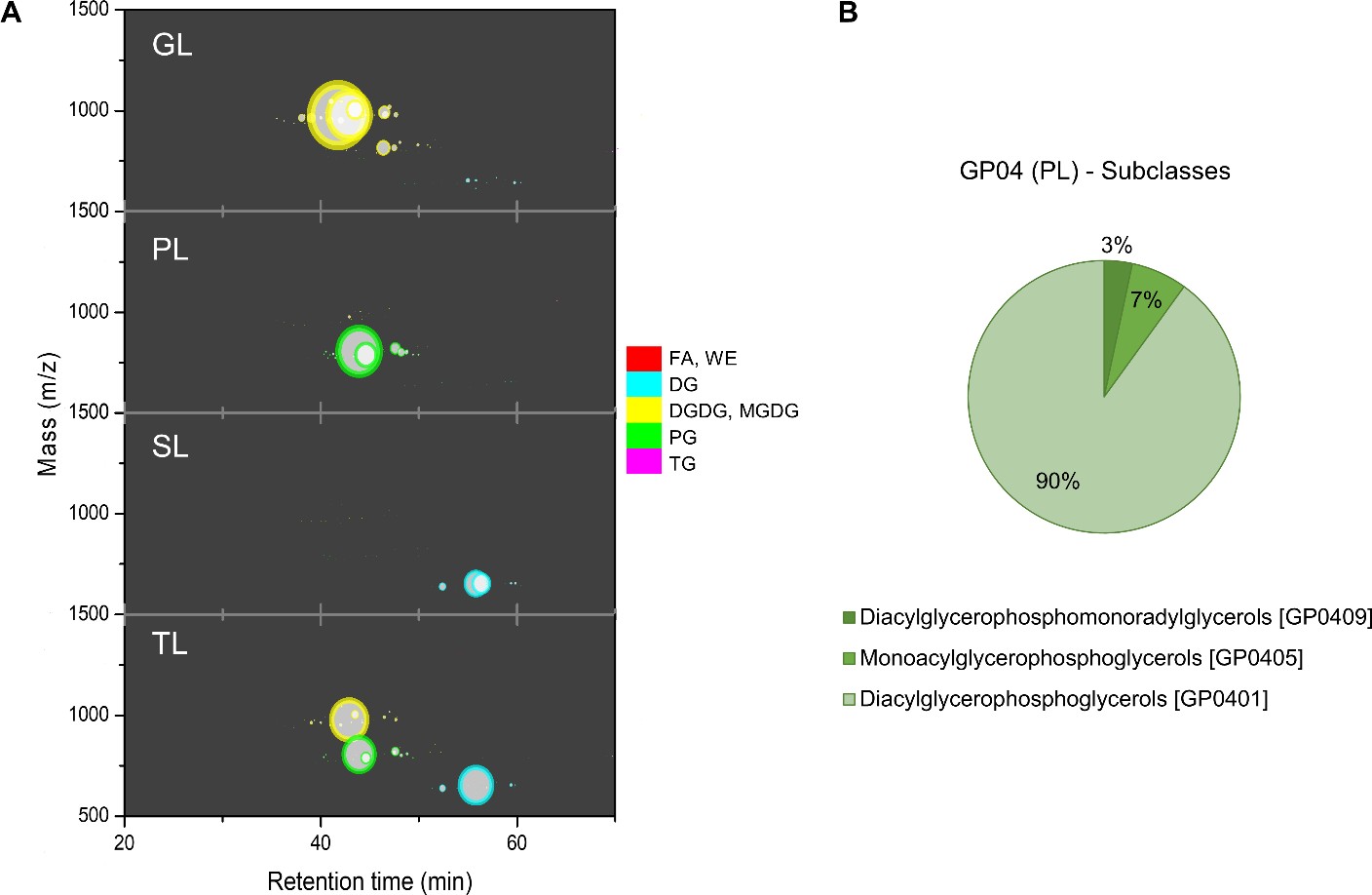


**Figure S7. Global lipidomics analysis of *Lactobacillus johnsonii* N6.2 total (TL) and fractionated lipids (SL, GL and PL) as determined by qualitative LC-MS/MS Analysis and MS-2 annotations.** A: Survey overview of lipid abundances from TL and lipid fractions detected in positive and negative modes (SL: simple lipids, GL: glycolipids, PL: phospholipids). B: Phospholipid subclasses identified in the PL fraction.


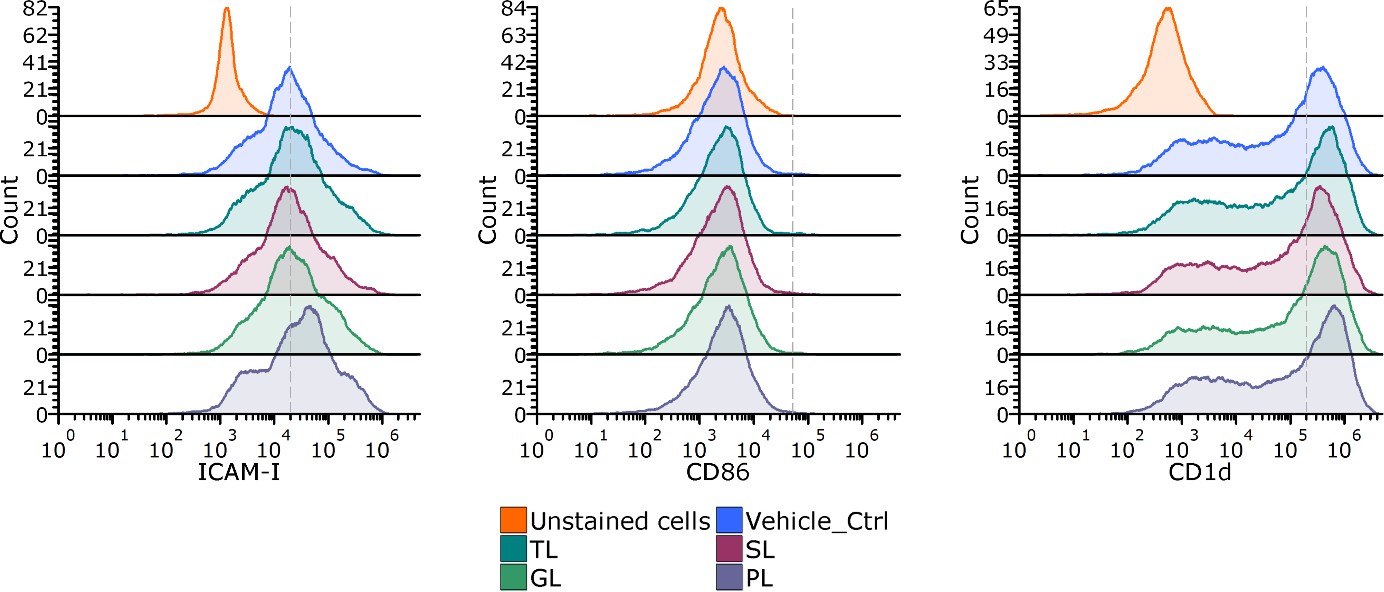


**Figure S8. Histogram overlays of significant surface marker expression changes in BMDCs stimulated with *Lactobacillus johnsonii* N6.2 total (TL) and fractionated lipids (SL, GL and PL).** The dotted line represents the median fluorescence intensity for the vehicle control. SL: simple lipids, GL: glycolipids, PL: phospholipids. Data is representative of three independent assays.


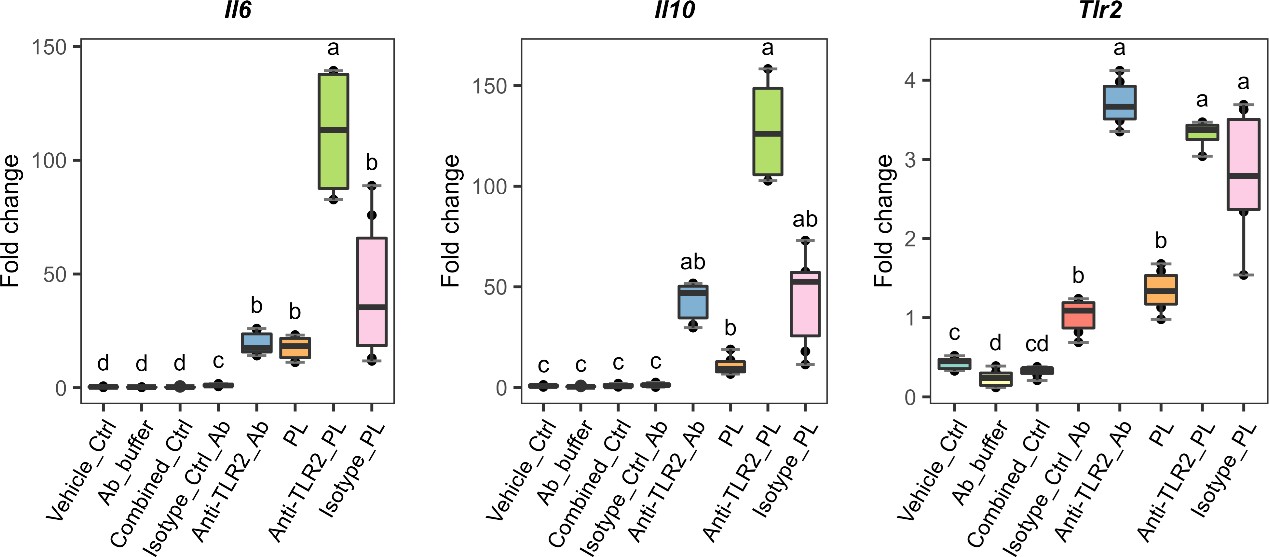


**Figure S9. Blocking canonical NF-kB signaling in *Lactobacillus johnsonii* N6.2 lipid- stimulated BMDCs.** 7-day BMDCs were pre incubated with the IKKβ kinase inhibitor IMD- 0354 at 0.1, 0.25 and 0.5 µM for 1h before addition of *Lj-*N6.2 total lipids (TLs) or phospholipids (PLs) at 0.5 or 5 µg/mL respectively and further incubated for 6 h. Negative controls: vehicle control for *Lj-*N6.2 lipids mixed with vehicle control for inhibitors (VC_combined), NF- kB inhibitor alone. Data were obtained from three independent assays. Different letter labels denote statistically significant changes (p.value < 0.05).


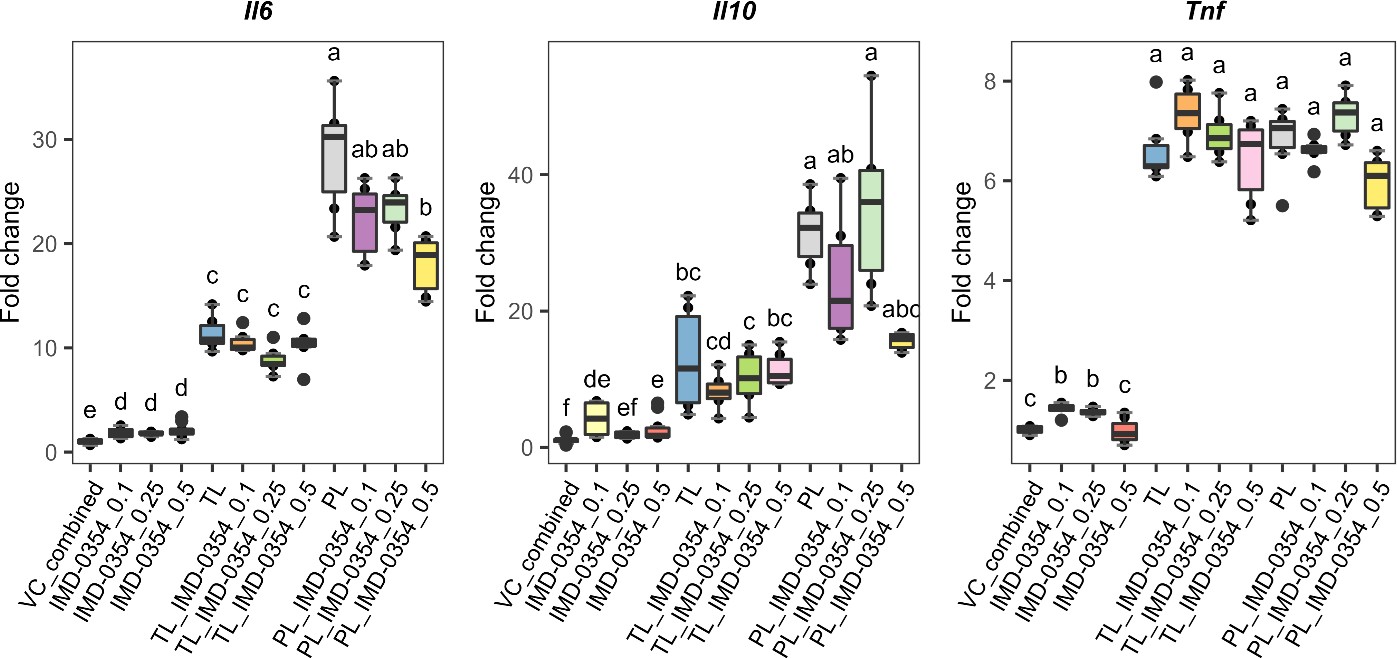


**Figure S10. Blocking canonical NF-kB signaling in *Lactobacillus johnsonii* N6.2 lipid- stimulated BMDCs.** 7-day BMDCs were pre incubated with the IKKβ kinase inhibitor IMD- 0354 at 0.1, 0.25 and 0.5 µM for 1h before addition of *Lj-*N6.2 total lipids (TLs) or phospholipids (PLs) at 0.5 or 5 µg/mL respectively and further incubated for 6 h. Negative controls: vehicle control for *Lj-*N6.2 lipids mixed with vehicle control for inhibitors (VC_combined), NF- kB inhibitor alone. Data were obtained from three independent assays. Different letter labels denote statistically significant changes (p.value < 0.05).


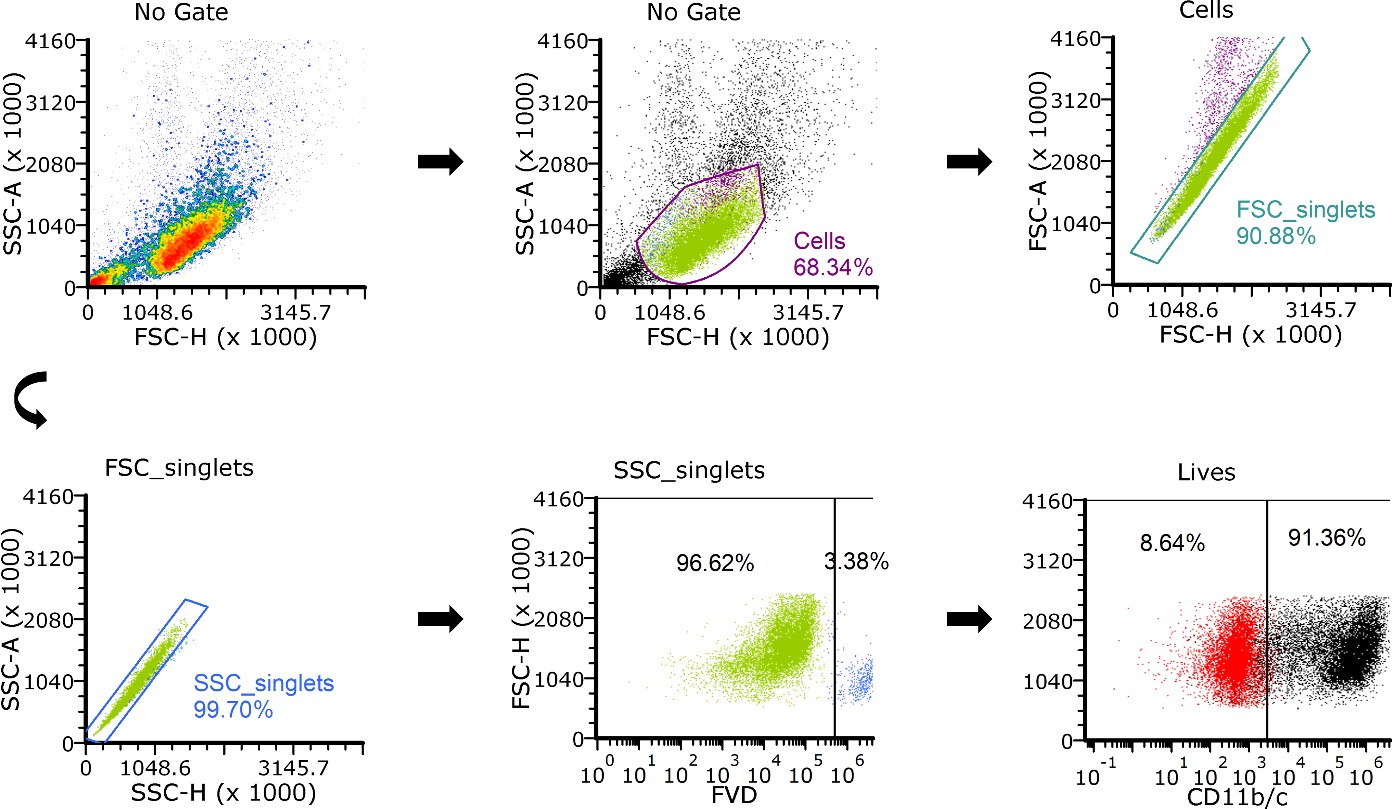


**Figure S11. Gating strategy and purity of BMDC cultures determined by flow cytometry.** The figure presents the gating strategy used to analyze data obtained by flow cytometry starting from doublet exclusion, cell viability evaluation (FVD), and positivity for surface CD11b/c. The green color throughout the panels represents live cells, and the red color in the last panel represents unstained (gated) cells. The Figure is representative of a routine purity evaluation.

Purity of non-adherent BMDCs as CD11b/c^+^ cells was consistently > 90%.

**Table S5. Primers used for qRT-PCR analysis.** *Gapdh* primers were sourced from Zhou et al.^1^ Additional primers were designed for this work.

| **Gene** | **Forward sequence (5'-3')** | **Reverse sequence (5'-3')** |
| --- | --- | --- |
| *Gapdh* | ATGACTCTACCCACGGCAAG | GGAAGATGGTGATGGGTTTC |
| *Il6* | ATTCTGTCTCGAGCCCACCAG | ACTGGCTGGAAGTCTCTTGCG |
| *Il10* | CTGGTAGAAGTGATGCCCCA | GGAGAAATCGATGACAGCGT |
| *Tlr2* | GCCTTATGGAAACTGCAAAGAGTCA | GCAGAATGGCCTTCCCTTGA |
| *Tlr4* | TCTGAGCTTCAACCCCCTGA | GCCATGCCATGCCTTGTCTT |
| *Adora2a* | GAGCAGCGCTAGTTTCGAAGT | CAGGGTTCTGAGATACCTCTTGC |
| *Cyp2s1* | ACCCTCAAGTCCAAAAGCGTG | AACAGCATCCGTGTAAGGGAG |
| *Tnf* | TCCCAACAAGGAGGAGAAGTTCC | CGGGCTTGTCACTCGAGTTTT |

# References

1. Zhou J, Ando H, Macova M, Dou J, Saavedra JM. Angiotensin II AT1 receptor blockade abolishes brain microvascular inflammation and heat shock protein responses in hypertensive rats. Journal of Cerebral Blood Flow and Metabolism. 2005;25(7):878-886. doi:10.1038/SJ.JCBFM.9600082/ASSET/IMAGES/LARGE/10.1038_SJ.JCBFM. 9600082-FIG8.JPEG
