# Supplementary material for: Lactobacillus johnsonii N6.2 phospholipids induce immature-like dendritic cells with a migratory-regulatory-like transcriptional signature
Source: Gut Microbes. 2023 Sep 7;15(2):2252447. doi: 10.1080/19490976.2023.2252447 (PMC10486300; doi:10.1080/19490976.2023.2252447)
Supplement: Supplemental Material [file KGMI_A_2252447_SM2693.zip › Supplementary material/Table S1.docx]

**Table S1. *Lactobacillus johnsonii* N6.2 total lipid profiling by qualitative lipidomic LC-MS/MS analysis.** The MS-2 annotations (precursor mass- and spectral-matched) detected are presented. RT = retention time. m/z = mass-to-charge ratio. Category: GCL* = Glycerolipid; GP = glycerophospholipid; FA = fatty acyl.

| **RT** | **m/z** | **Name_matched** | **Category** |
| --- | --- | --- | --- |
| 45.923 | 775.5492 | _1-_2,3-dihydroxypropoxy_-hydroxyphosphoryl_oxy-3-_octadec-11-enoyl_oxypropan-2-yl_ octadecanoate | FA |
| 46.559 | 775.5487 | _2.3-dihydroxypropoxy_3-_octadec-9-enoyloxy_-2-_octadecanoyloxy_propoxy_phosphinic acid | FA |
| 11.87 | 323.2558 | _R_-2-hydroxystearic acid | FA |
| 54.384 | 299.258 | _R_-2-hydroxystearic acid | FA |
| 55.82 | 614.5721 | DG 16:0_18:0 | GCL |
| 55.211 | 626.5721 | DG 16:0_19:1 | GCL |
| 59.779 | 642.6041 | DG 18:0_18:0 | GCL |
| 60.445 | 642.6039 | DG 18:0_18:0 | GCL |
| 56.956 | 640.5881 | DG 18:0_18:1 | GCL |
| 59.42 | 654.6034 | DG 18:0_19:1 | GCL |
| 59.851 | 654.6047 | DG 18:0_19:1 | GCL |
| 58.839 | 654.6035 | DG 18:0_19:1 | GCL |
| 52.422 | 638.5722 | DG 18:1_18:1 | GCL |
| 53.907 | 638.5729 | DG 18:1_18:1 | GCL |
| 51.378 | 638.5725 | DG 18:1_18:1 | GCL |
| 49.641 | 636.5563 | DG 18:1_18:2 | GCL |
| 48.604 | 636.5566 | DG 18:1_18:2 | GCL |
| 55.857 | 652.5874 | DG 18:1_19:1 | GCL |
| 55.024 | 652.5879 | DG 18:1_19:1 | GCL |
| 56.388 | 652.5882 | DG 18:1_19:1 | GCL |
| 52.368 | 650.572 | DG 19:1_18:2 | GCL |
| 57.94 | 666.6035 | DG 19:1_19:1 | GCL |
| 38.421 | 936.662 | DGDG 16:0_18:1 | GCL |
| 37.319 | 936.6635 | DGDG 16:0_18:1 | GCL |
| 42.086 | 950.6778 | DGDG 16:0_19:1 | GCL |
| 40.996 | 950.679 | DGDG 16:0_19:1 | GCL |
| 37.317 | 948.6623 | DGDG 16:1_19:1 | GCL |
| 36.313 | 948.6639 | DGDG 16:1_19:1 | GCL |
| 44.236 | 964.6941 | DGDG 17:0_19:1 | GCL |
| 43.154 | 964.6953 | DGDG 17:0_19:1 | GCL |
| 44.97 | 991.6573 | DGDG 18:0_18:1 | GCL |
| 47.678 | 978.7096 | DGDG 18:0_19:1 | GCL |
| 46.614 | 978.7107 | DGDG 18:0_19:1 | GCL |
| 39.085 | 962.6775 | DGDG 18:1_18:1 | GCL |
| 40.046 | 962.6771 | DGDG 18:1_18:1 | GCL |
| 40.505 | 989.6423 | DGDG 18:1_18:1 | GCL |
| 41.147 | 989.6412 | DGDG 18:1_18:1 | GCL |
| 40.497 | 962.6808 | DGDG 18:1_18:1 | GCL |
| 38.093 | 962.6788 | DGDG 18:1_18:1 | GCL |
| 35.558 | 960.6641 | DGDG 18:1_18:2 | GCL |
| 42.915 | 976.6931 | DGDG 18:1_19:1 | GCL |
| 43.529 | 1003.657 | DGDG 18:1_19:1 | GCL |
| 44.243 | 976.6953 | DGDG 18:1_19:1 | GCL |
| 44.071 | 1003.658 | DGDG 18:1_19:1 | GCL |
| 44.594 | 976.6935 | DGDG 18:1_19:1 | GCL |
| 45.484 | 976.6938 | DGDG 18:1_19:1 | GCL |
| 72.714 | 976.6943 | DGDG 18:1_19:1 | GCL |
| 41.789 | 976.6942 | DGDG 18:1_19:1 | GCL |
| 39.388 | 974.6782 | DGDG 19:1_18:2 | GCL |
| 38.36 | 974.6797 | DGDG 19:1_18:2 | GCL |
| 46.504 | 990.7098 | DGDG 19:1_19:1 | GCL |
| 47.018 | 1017.673 | DGDG 19:1_19:1 | GCL |
| 47.844 | 990.711 | DGDG 19:1_19:1 | GCL |
| 42.143 | 1044.793 | DGDG O-17:2_26:1 | GCL |
| 41.101 | 1044.794 | DGDG O-17:2_26:1 | GCL |
| 64.124 | 1056.823 | HBMP 18:1_18:1_18:1 | GP |
| 12.48 | 509.2878 | LPG 18:1 | GP |
| 13.922 | 523.3036 | LPG 19:1 | GP |
| 46.783 | 788.6245 | MGDG 16:0_19:1 | GCL |
| 45.7 | 788.6253 | MGDG 16:0_19:1 | GCL |
| 51.172 | 816.6569 | MGDG 18:0_19:1 | GCL |
| 52.248 | 816.6563 | MGDG 18:0_19:1 | GCL |
| 42.645 | 800.6258 | MGDG 18:1_18:1 | GCL |
| 43.62 | 800.624 | MGDG 18:1_18:1 | GCL |
| 47.514 | 814.6407 | MGDG 18:1_19:1 | GCL |
| 48.1 | 841.6041 | MGDG 18:1_19:1 | GCL |
| 46.411 | 814.6414 | MGDG 18:1_19:1 | GCL |
| 50.926 | 828.6569 | MGDG 19:1_19:1 | GCL |
| 51.671 | 855.6199 | MGDG 19:1_19:1 | GCL |
| 49.922 | 828.6572 | MGDG 19:1_19:1 | GCL |
| 18.167 | 255.2329 | Palmitic acid | FA |
| 43.363 | 780.5714 | PG 16:0_19:1 | GP |
| 43.914 | 761.533 | PG 16:0_19:1 | GP |
| 44.439 | 761.5344 | PG 16:0_19:1 | GP |
| 39.606 | 759.5174 | PG 16:1_19:1 | GP |
| 45.312 | 794.5908 | PG 18:0_18:1 | GP |
| 48.826 | 808.606 | PG 18:0_19:1 | GP |
| 49.432 | 789.5646 | PG 18:0_19:1 | GP |
| 49.994 | 789.564 | PG 18:0_19:1 | GP |
| 40.332 | 792.5746 | PG 18:1_18:1 | GP |
| 41.299 | 792.5741 | PG 18:1_18:1 | GP |
| 43.931 | 806.5899 | PG 18:1_19:1 | GP |
| 44.641 | 787.5487 | PG 18:1_19:1 | GP |
| 46.531 | 806.5901 | PG 18:1_19:1 | GP |
| 48.042 | 787.5488 | PG 18:1_19:1 | GP |
| 56.492 | 787.5481 | PG 18:1_19:1 | GP |
| 77.784 | 787.5487 | PG 18:1_19:1 | GP |
| 61.169 | 787.5466 | PG 18:1_19:1 | GP |
| 40.568 | 804.5741 | PG 19:1_18:2 | GP |
| 40.989 | 787.5493 | PG 19:1_18:2 | GP |
| 41.639 | 785.5331 | PG 19:1_18:2 | GP |
| 47.618 | 820.6061 | PG 19:1_19:1 | GP |
| 48.23 | 801.5642 | PG 19:1_19:1 | GP |
| 48.759 | 801.5657 | PG 19:1_19:1 | GP |
| 41.533 | 773.5331 | PG 36:2 | GP |
| 40.754 | 773.5333 | PG 36:2 | GP |
| 42.317 | 773.5317 | PG 36:2 | GP |
| 38.728 | 771.5185 | PG 36:3 | GP |
| 69.761 | 796.7391 | TG 14:0_16:0_16:0 | GCL |
| 69.135 | 796.7375 | TG 14:0_16:0_16:0 | GCL |
| 70.312 | 810.7544 | TG 15:0_16:0_16:0 | GCL |
| 71.137 | 824.771 | TG 16:0_16:0_16:0 | GCL |
| 70.057 | 848.7708 | TG 16:0_16:1_18:1 | GCL |
| 73.579 | 880.8344 | TG 16:0_18:0_18:0 | GCL |
| 71.409 | 876.8028 | TG 16:0_18:1_18:1 | GCL |
| 74.538 | 908.8654 | TG 18:0_18:0_18:0 | GCL |

*: The abbreviation GCL for glycerolipids was selected to avoid confusion with GL (denotation for the glycolipid fraction obtained after lipid fractionation of *L. johnsonii* N6.2 total lipids).
